# 104-week efficacy and safety of cipaglucosidase alfa plus miglustat in adults with late-onset Pompe disease: a phase III open-label extension study (ATB200-07)

**DOI:** 10.1007/s00415-024-12236-0

**Published:** 2024-02-28

**Authors:** Benedikt Schoser, Priya S. Kishnani, Drago Bratkovic, Barry J. Byrne, Kristl G. Claeys, Jordi Díaz-Manera, Pascal Laforêt, Mark Roberts, Antonio Toscano, Ans T. van der Ploeg, Jeff Castelli, Mitchell Goldman, Fred Holdbrook, Sheela Sitaraman Das, Yasmine Wasfi, Tahseen Mozaffar, the ATB200-07 Study Group, Agnes Sebok, Alan Pestronk, Aleksandra Dominovic-Kovacevic, Aneal Khan, Blaž Koritnik, Celine Tard, Christopher Lindberg, Colin Quinn, Crystal Eldridge, Cynthia Bodkin, David Reyes-Leiva, Derralynn Hughes, Ela Stefanescu, Emmanuelle Salort-Campana, Ernest Butler, Francoise Bouhour, Gee Kim, George Konstantinos Papadimas, Giancarlo Parenti, Halina Bartosik-Psujek, Hani Kushlaf, Hashiguchi Akihiro, Heather Lau, Helio Pedro, Henning Andersen, Hernan Amartino, Hideaki Shiraishi, Hiroshi Kobayashi, Ivaylo Tarnev, Jaime Vengoechea, Jennifer Avelar, Jin-Hong Shin, John Nevin, Jonathan Cauci, Jorge Alonso-Pérez, Jozsef Janszky, Julie Berthy, Cornelia Kornblum, Kristina Gutschmidt, Maria Judit Molnar, Marie Wencel, Mark Tarnopolsky, Matthias Boentert, Michel Tchan, Miriam Freimer, Nicola Longo, Nicolas Abreu, Nuria Vidal-Fernandez, Olimpia Musumeci, Ozlem Goker-Alpan, Patrick Deegan, Paula R. Clemens, Richard Roxburgh, Robert Henderson, Robert Hopkin, Sabrina Sacconi, Simona Fecarotta, Shahram Attarian, Stephan Wenninger, Stephanie Dearmey, Tarekegn Hiwot, Thomas Burrow, Tobias Ruck, Tomo Sawada, Vescei Laszlo, Wolfgang Löscher, Yin-Hsiu Chien

**Affiliations:** 1 Friedrich-Baur-Institute at the Department of Neurology, LMU University Hospital, LMU Munich, Munich, Germany; 2 Duke University Medical Center, Durham, NC USA; 3 PARC Research Clinic, Royal Adelaide Hospital, Adelaide, SA Australia; 4 University of Florida, Gainesville, FL USA; 5 Department of Neurology, University Hospitals Leuven, Leuven, Belgium; 6 Laboratory for Muscle Diseases and Neuropathies, Department of Neurosciences, KU Leuven, Leuven, Belgium; 7 John Walton Muscular Dystrophy Research Centre, Newcastle University International Centre for Life, Newcastle Upon Tyne, UK; 8 Neurology Department, Nord/Est/Île-de-France Neuromuscular Reference Center, FHU PHENIX, Raymond-Poincaré Hospital, AP-HP, Garches, France; 9 Salford Royal NHS Foundation Trust, Salford, UK; 10 ERN-NMD Center for Neuromuscular Disorders of Messina, Department of Clinical and Experimental Medicine, University of Messina, Messina, Italy; 11 Erasmus MC University Medical Center, Rotterdam, Netherlands; 12 Amicus Therapeutics, Inc, Princeton, NJ USA; 13 Department of Neurology, University of California, Irvine, CA USA

**Keywords:** Glycogen storage disease type II, Alpha glucosidase, Myozyme, *n*-butyldeoxynojirimycin, Lysosomal storage disorders

## Abstract

The phase III double-blind PROPEL study compared the novel two-component therapy cipaglucosidase alfa + miglustat (cipa + mig) with alglucosidase alfa + placebo (alg + pbo) in adults with late-onset Pompe disease (LOPD). This ongoing open-label extension (OLE; NCT04138277) evaluates long-term safety and efficacy of cipa + mig. Outcomes include 6-min walk distance (6MWD), forced vital capacity (FVC), creatine kinase (CK) and hexose tetrasaccharide (Hex4) levels, patient-reported outcomes and safety. Data are reported as change from PROPEL baseline to OLE week 52 (104 weeks post-PROPEL baseline). Of 118 patients treated in the OLE, 81 continued cipa + mig treatment from PROPEL (cipa + mig group; 61 enzyme replacement therapy [ERT] experienced prior to PROPEL; 20 ERT naïve) and 37 switched from alg + pbo to cipa + mig (switch group; 29 ERT experienced; 8 ERT naive). Mean (standard deviation [SD]) change in % predicted 6MWD from baseline to week 104 was + 3.1 (8.1) for cipa + mig and − 0.5 (7.8) for the ERT-experienced switch group, and + 8.6 (8.6) for cipa + mig and + 8.9 (11.7) for the ERT-naïve switch group. Mean (SD) change in % predicted FVC was − 0.6 (7.5) for cipa + mig and − 3.8 (6.2) for the ERT-experienced switch group, and − 4.8 (6.5) and − 3.1 (6.7), respectively, in ERT-naïve patients. CK and Hex4 levels improved in both treatment groups by week 104 with cipa + mig treatment. Three patients discontinued the OLE due to infusion-associated reactions. No new safety signals were identified. Cipa + mig treatment up to 104 weeks was associated with overall maintained improvements (6MWD, biomarkers) or stabilization (FVC) from baseline with continued durability, and was well tolerated, supporting long-term benefits for patients with LOPD.

**Trial registration number**: NCT04138277; trial start date: December 18, 2019.

**Supplementary Information:**

The online version contains supplementary material available at 10.1007/s00415-024-12236-0.

## Introduction

Pompe disease is a rare, inherited, multisystemic and progressive lysosomal disorder caused by biallelic pathogenic variants in the acid α-glucosidase (*GAA*) gene, resulting in a functional deficiency of GAA enzyme [1–3]. The impaired function of GAA leads to the accumulation of lysosomal glycogen in muscle, which causes dysregulated autophagy, disrupting muscle architecture and irreversible damage to skeletal, cardiac and smooth muscles [1, 4]. Pompe disease is considered a wide spectrum of phenotypes. Patients with the most severe phenotype, infantile-onset Pompe disease (IOPD), usually have < 1% residual GAA enzyme activity and typically develop symptoms, including rapid and progressive loss of muscle function and strength, hypertrophic cardiomyopathy, and death from respiratory failure in the first 2 years of life if left untreated. Patients with late-onset Pompe disease (LOPD) usually have 1–30% residual GAA activity and may develop symptoms at any age [1, 5–8]. Most patients with LOPD initially experience progressive loss of skeletal muscle function, usually starting with the axial and proximal muscles (trunk and lower limbs), followed by involvement of the proximal upper limbs and diaphragm, resulting in respiratory insufficiency. Over time, symptoms often lead to a need for wheelchair use and assisted ventilation [7, 9, 10].

Enzyme replacement therapy (ERT) with alglucosidase alfa, a recombinant human GAA (rhGAA), was the first approved treatment for Pompe disease [5, 11–14]. For many patients with LOPD, ERT with alglucosidase alfa initially leads to a slowing of disease progression followed by a decline in efficacy after 3–5 years, highlighting a critical unmet need for new therapies with a more durable response [15–18]. The variable and suboptimal long-term efficacy of alglucosidase alfa in LOPD prompted further research to better understand the challenges in delivering rhGAA to the lysosome of skeletal muscle. To date, three key challenges have been described in the literature: (1) despite large amounts of rhGAA infused into the blood (≥ 20 mg/kg), only a small percentage reaches the skeletal muscle due in part to clearance in the liver, suggesting that high-affinity binding to the cation-independent mannose 6-phosphate receptor (CI-MPR) is required for uptake of the remaining ERT into target muscle cells [19, 20]; (2) rhGAA is delivered to the target tissue as a precursor that requires both proteolytic and N-glycan trimming to be converted into the version of GAA with the highest enzyme activity toward glycogen (7–10 × the activity of the precursor protein) [21]; (3) rhGAA is relatively unstable at the near-neutral pH of the blood and is rapidly inactivated following infusion [19].

Cipaglucosidase alfa plus miglustat (cipa + mig) is a novel two-component therapy designed to address the key challenges outlined above to improve rhGAA delivery to skeletal muscle lysosomes [19, 21, 22]. Cipaglucosidase alfa is enriched with Chinese hamster ovary (CHO)-cell (naturally) derived bis-phosphorylated mannose-6-phosphate (bis-M6P) N-glycans to mediate high-affinity binding and effective uptake into muscle via CI-MPR while retaining its capacity for intracellular processing [20, 21]. Inside the cell, cipaglucosidase alfa undergoes proteolytic and N-glycan processing into the fully processed mature form of the enzyme with maximal catalytic activity [20–22]. The oral enzyme stabilizer miglustat binds to and stabilizes cipaglucosidase alfa in the bloodstream thus minimizing inactivation and increasing the amount of rhGAA available for targeting to skeletal muscle [20, 21, 23]. In pre-clinical studies, cipa + mig improved multiple defects along the Pompe disease pathogenic cascade in *GAA* knockout mice, including reduced lysosomal enlargement and autophagic build-up, resulting in improved muscle quality, architecture and strength compared with alglucosidase alfa-treated or untreated mice [20, 24].

The pivotal phase III PROPEL study (ATB200-03; NCT03729362) compared the efficacy and safety of cipa + mig versus standard-of-care ERT alglucosidase alfa in ambulatory adults with LOPD over 52 weeks [25]. Unlike other phase III studies in LOPD, PROPEL included a majority of patients previously treated with alglucosidase alfa (mean > 7 years treatment duration), reflecting the real-world treated LOPD patient population [26]. While the primary endpoint (change from baseline to week 52 in 6-min walk distance [6MWD] in meters) showed a mean improvement for cipa + mig versus alglucosidase alfa in the overall population, this difference was not statistically significant for superiority. Therefore, subsequent analyses of secondary endpoints were interpreted as nominal statistical assessments of superiority. Nominally statistically significant improvements versus alglucosidase alfa were seen at week 52 in the overall population of PROPEL for respiratory function (forced vital capacity [FVC]) and biomarker levels [25]. Additionally, nominally significant improvements were reached in the largest pre-specified population (the ERT-experienced group) for both 6MWD and FVC. Here, we describe data from 2 years of treatment with cipa + mig in the PROPEL study (ATB200-03), plus the ongoing open-label extension (OLE) of PROPEL (ATB200-07), which aims to assess the long-term efficacy and safety of cipa + mig.

## Methods

### Study design

Study ATB200-07 (NCT04138277) is an ongoing OLE of the randomized, double-blind, phase III study ATB200-03 (NCT03729362; PROPEL) [25]. The study is being conducted at 60 sites in 23 countries (Argentina, Australia, Austria, Belgium, Bosnia, Canada, Denmark, France, Germany, Greece, Hungary, Italy, Japan, South Korea, the Netherlands, New Zealand, Poland, Slovenia, Spain, Sweden, Taiwan, the United Kingdom, and the United States). The first patient was enrolled in December 2019. This manuscript describes data from the first year of treatment with cipa + mig in the OLE study (data cutoff date January 2022). For patients who were treated with cipa + mig during PROPEL, the data include a total of 2 years of treatment with cipa + mig, whereas for patients who were treated with alglucosidase alfa plus placebo (alg + pbo) in PROPEL, the data presented focus on their first year of treatment with cipa + mig in study ATB200-07 after switching from alg + pbo.

### Study participants

The main inclusion criterion for the OLE was the completion of PROPEL. All patients must have provided written, informed consent for the OLE. Female patients of childbearing potential and male patients must have agreed to use medically acceptable forms of contraception during the study and for 90 days after the last dose of study treatment. Patients were excluded from the study if they received gene therapy or participated in another interventional study for Pompe disease. Other exclusion criteria were hypersensitivity to the excipients in cipa + mig and any medical conditions or other extenuating circumstances that could pose an undue safety risk to the patient or may have compromised their ability to comply with or adversely impacted protocol requirements. Pregnant or breastfeeding patients or those planning to conceive a child during the study were also excluded.

### Treatments

Treatment protocols during PROPEL were previously outlined [25]. All patients received combination treatment with cipa + mig every 2 weeks. The first infusion visit in the OLE was scheduled ~ 2 weeks after the last study drug administration in PROPEL. Miglustat was administered as 65 mg oral capsules (3 capsules [195 mg total] for patients with a body weight of ≥ 40 kg and < 50 kg, or 4 capsules [260 mg total] for patients with a body weight of ≥ 50 kg). Patients had to fast for ≥ 2 h before and after taking miglustat. Cipaglucosidase alfa was administered intravenously over approximately 4 h, starting ~ 1 h after the administration of miglustat, at a dose of 20 mg/kg body weight. Study drugs were administered in a hospital/clinic setting for the first 3 months of the study. Patients who did not have any moderate or severe infusion-associated reaction (IAR) during this time may have been eligible for treatment with the study drug in their home (in countries where the administration of standard ERT with alglucosidase alfa was not reserved for the hospital/clinic setting).

### Assessments and outcomes

#### Efficacy

Efficacy assessments included motor function tests (6MWD; Gait, Stairs, Gowers’ maneuver and Chair [GSGC]), pulmonary function tests (sitting FVC), muscle strength tests (manual muscle testing [MMT] for the lower extremities), and patient-reported outcomes (PROs), including the Patient-Reported Outcomes Measurement Information System (PROMIS)–Physical Function Short Form (SF) 20a and PROMIS–Fatigue SF 8a. 6MWD and FVC are presented as % predicted. This calculation standardizes the actual distance walked in meters by the predicted value of a healthy person of comparable gender, age, height and weight for 6MWD [27]. For FVC, values are compared to those of a healthy person of comparable gender, age, height and race [28]. Tests were administered, where possible, by the same person at each visit with the identity of the test administrator being recorded. Training was provided by a central vendor to limit interobserver variability across the study. Assessments were conducted at the OLE baseline (for patients who had missed assessments at week 52 in PROPEL), at OLE week 12, OLE week 26, and then every 26 weeks thereafter. Laboratory assessments, including serum creatine kinase (CK) levels and urine hexose tetrasaccharide (Hex4) levels were assessed at the OLE baseline, at OLE weeks 2, 4, 6, 12 and 26, and every 26 weeks thereafter. Data through OLE week 52 are presented.

#### Safety

Safety assessments were performed throughout and included monitoring for treatment-emergent adverse events (TEAEs), serious TEAEs and IARs, clinical laboratory profiles (serum chemistry, hematology, and urinalysis), vital signs, physical examinations, and immunogenicity. Preferred terms of adverse events were coded with MedDRA Version 23.0.

#### Immunogenicity

Immunogenicity endpoints included total anti-drug antibodies (ADAs), including titers and neutralizing antibodies (NAbs) for enzyme activity (inhibition of rhGAA-mediated hydrolysis of 4-methylumbelliferyl-glucoside, inhibition of rhGAA-mediated hydrolysis of glycogen) and enzyme uptake (inhibition of rhGAA binding to CI-MPR). Blood samples for measurement of anti-rhGAA antibodies (total, cross-reactive, and neutralizing) and total GAA protein concentration were collected pre-dose, at time points up to 130 weeks after the PROPEL baseline. Total GAA protein concentration was measured from the same blood sample, as assay sensitivity for anti-rhGAA antibodies and IgE can be affected by GAA protein levels.

### Data analysis

This single-arm OLE study had no control group. In the pivotal PROPEL study, 6MWD was specified as the primary endpoint. However, in this OLE, no efficacy endpoint is designated as ‘primary,’ and there were no formal hypotheses. Continuous variables were summarized using descriptive statistics (*n*, mean, standard deviation [SD], median, first quartile, third quartile, minimum, and maximum); categorical variables were summarized using number and percentage. A 95% confidence interval for the mean difference was calculated for summaries involving the change from baseline. No formal sample size calculation was conducted.

Efficacy endpoints were analyzed in the OLE-enrolled subjects (OLE-ES) population, which included all patients eligible for and enrolled in the OLE, regardless of whether or not they received any study drug. The safety population included all patients who received at least one dose of the study drug in PROPEL or the OLE.

Data were analyzed in treatment groups based on treatment received in PROPEL. The cipa + mig group included patients who had been randomized to cipa + mig in PROPEL and continued this treatment in the OLE. The switch group included patients randomized to alg + pbo in PROPEL and switched to cipa + mig in the OLE.

## Results

### Patient disposition

**Fig. 1:**
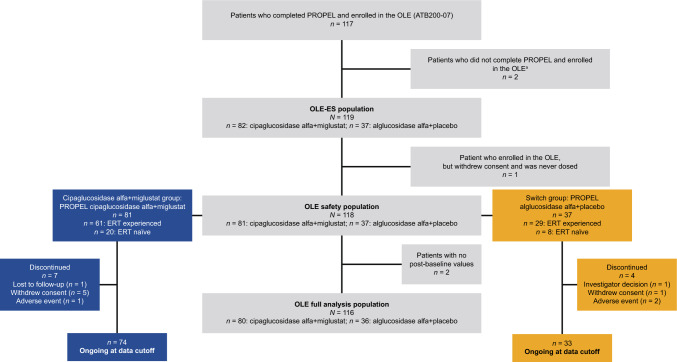
Patient disposition. ^a^Two patients who withdrew from PROPEL for logistical reasons not related to the efficacy or safety of cipa + mig (one due to an AE of COVID-19-related pneumonia and one because of concerns about travelling to the study site due to COVID-19) were enrolled in the OLE. *AE* adverse event; *ERT* enzyme replacement therapy; *OLE* open-label extension; *OLE-ES* open-label extension enrolled subjects

Of the 123 patients enrolled in PROPEL, 117 completed the study and continued in the OLE. In addition, two patients who withdrew from PROPEL for logistical reasons not related to the efficacy or safety of cipa + mig (one due to COVID-19-related pneumonia and one because of concerns about traveling to the study site due to COVID-19) were enrolled in the OLE. Hence, the OLE enrolled a total of 119 patients (91 ERT experienced prior to PROPEL and 28 ERT naïve; Fig. 1). However, as one patient withdrew consent and did not receive any study treatment in ATB200-07, the final number of patients who received treatment in the OLE was 118. As previously described, one ERT-naïve patient in the alglucosidase alfa group in the PROPEL study was deemed by the principal investigator as likely to have deliberately underperformed at baseline and this outlier patient was excluded from the efficacy analyses in PROPEL [25] and the efficacy analysis presented here. 107 patients (90.7%) were still ongoing at the data cutoff (January 11, 2022; Fig. 1).

### Patient demographics and baseline characteristics

In line with PROPEL, baseline characteristics were representative of the population of patients with LOPD [25] and similar between treatment groups (Table 1). Approximately three-quarters of patients (76.3%) were ERT experienced prior to entering PROPEL (61 in the cip + mig group and 29 in the switch group). Among these, the mean ERT duration was 7.3 years (range: 2–14 years) and 66.7% had received alglucosidase alfa for 5 years or more. A smaller subset of patients in PROPEL (23.7%) had never received ERT (ERT naïve; 20 in the cipa + mig group and 8 in the switch group). During PROPEL, 28 patients on cipa + mig and 11 patients on alg + pbo received at least one administration of study drugs at home; this increased to 67 patients in the OLE.

**Table 1:** Demographics and baseline characteristics (OLE-safety population)

	Cipa + mig group (*N* = 81)	Switch group (*N* = 37)	Total (*N* = 118)
Age at informed consent date, years			
Mean (SD)	48.9 (13.5)	46.0 (13.5)	48.0 (13.5)
Median (range)	49.0 (20–75)	47.0 (23–67)	49.0 (20–75)
Age at diagnosis, years			
Mean (SD)	40.3 (13.8)	37.2 (15.4)	39.3 (14.4)
Median (range)	40.0 (1–66)	40.0 (7–63)	40.0 (1–66)
Gender, *n* (%)			
Male	33 (40.7)	19 (51.4)	52 (44.1)
Female	48 (59.3)	18 (48.6)	66 (55.9)
Race, *n* (%)^a^			
Asian	3 (3.7)	1 (2.7)	4 (3.4)
Japanese	2 (2.5)	4 (10.8)	6 (5.1)
Black/African American	0 (0.0)	1 (2.7)	1 (0.8)
White	71 (87.7)	30 (81.1)	101 (85.6)
Other	5 (6.2)	1 (2.7)	6 (5.1)
Region,* n* (%)			
North/South America	24 (29.6)	14 (37.8)	38 (32.2)
Europe	42 (51.9)	12 (32.4)	54 (45.8)
Asia Pacific	15 (18.5)	11 (29.7)	26 (22.0)
Height, cm			
Mean (SD)	171.2 (9.7)	171.2 (11.3)^b^	171.2 (10.2)^c^
Weight, kg			
Mean (SD)	73.3 (15.3)	78.9 (26.8)	75.1 (19.7)
ERT status at entry into PROPEL, *n* (%)			
Naïve	20 (24.7)	8 (21.6)	28 (23.7)
Experienced	61 (75.3)	29 (78.4)	90 (76.3)
ERT duration prior to PROPEL,^d^ years			
Mean (SD)	7.5 (3.4)	7.0 (3.7)	7.3 (3.5)
Median (range)	7.6 (2–14)	7.1 (2–13)	7.4 (2–14)

*Cipa* + *mig* cipaglucosidase alfa + miglustat; *ERT* enzyme replacement therapy; *OLE* open-label extension; *SD* standard deviation

^a^Patients could choose more than one category

^b^*n* = 36

^c^*n* = 117

^d^ERT-experienced patients only

### Efficacy outcomes: ERT-experienced patient cohort

Results for both week 52 (the end of PROPEL) and week 104 (week 52 of the OLE) are reported as mean change from the baseline (CFBL) of PROPEL.

**Fig. 2:**
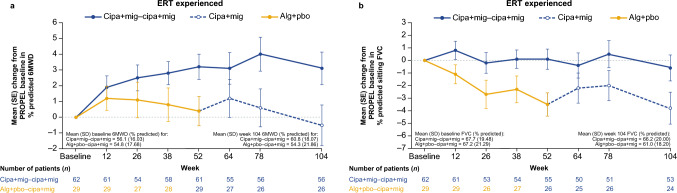
Change from baseline in **a** 6MWD (% predicted) and **b** sitting FVC (% predicted) in ERT-experienced patients (OLE-ES population). *6MWD* 6-min walk distance; *alg* + *pbo* alglucosidase alfa + placebo; *cipa* + *mig* cipaglucosidase alfa + miglustat; *ERT* enzyme replacement therapy; *FVC* forced vital capacity; *OLE-ES* open-label extension enrolled subjects; *SD* standard deviation; *SE* standard error

ERT-experienced patients treated with cipa + mig throughout PROPEL and the OLE showed increased % predicted 6MWD at week 104 (mean CFBL + 3.1 [SD 8.1]) compared with those in the switch group (mean CFBL −0.5 [SD 7.8]; Fig. 2a). Absolute 6MWD data in meters are summarized in Supplementary Table S1.

ERT-experienced patients in the cipa + mig group showed a numerical decrease in GSGC total score from baseline to week 104 (mean CFBL −0.9 [SD 2.6]; a decreased score being suggestive of an improvement in this measure). In contrast, in the switch group, after an initial increase during PROPEL (mean CFBL + 0.7 [SD 2.0]), patients’ GSGC total score remained the same after switching to cipa + mig in the OLE without any further progression from week 52 to week 104 (mean CFBL + 0.6 [SD 2.2]; Supplementary Fig. S1).

ERT-experienced patients in the cipa + mig group showed a numerical increase in MMT lower extremity score in PROPEL (mean CFBL + 1.6 [SD 4.3]; increased scores suggest improvement in this measure); scores were maintained at the same level from week 52 through week 104 (mean CFBL + 1.6 [SD 4.6]; Supplementary Fig. S2). In comparison, patients in the switch group showed a smaller increase in muscle strength scores in PROPEL (mean CFBL + 0.9 [SD 2.9]) but reached similar increases from baseline as the cipa + mig group after switching in the OLE (mean CFBL + 1.5 [SD 2.9]).

For lung function assessments, ERT-experienced patients in the cipa + mig group remained relatively stable in % predicted sitting FVC from baseline to week 104 (mean CFBL −0.6 [SD 7.5]; Fig. 2b). Patients in the switch group experienced a numerical decrease in % predicted sitting FVC during alg + pbo treatment in PROPEL to week 52 (mean CFBL −3.5 [SD 4.7]); this was maintained to a similar level to week 104 after switching to cipa + mig in the OLE (mean CFBL −3.8 [SD 6.2]).

**Fig. 3:**
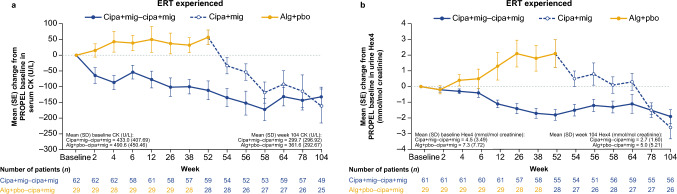
Change from baseline in **a** serum CK and **b** urine Hex4 levels in ERT-experienced patients (OLE-ES population). *Alg* + *pbo* alglucosidase alfa + placebo; *cipa* + *mig* cipaglucosidase alfa + miglustat; *CK* creatine kinase; *ERT* enzyme replacement therapy; *Hex4* hexose tetrasaccharide; *OLE-ES* open-label extension enrolled subjects; *SD* standard deviation; *SE* standard error

Overall, cipa + mig treatment led to decreased levels of serum CK and urine Hex4 in both the cipa + mig and switch groups (Fig. 3a, b). For ERT-experienced patients in the cipa + mig group, serum CK levels decreased from baseline to week 52 in PROPEL (mean CFBL −111.4 U/L [SD 229.0]) and remained stable throughout the OLE to week 104 (mean CFBL −132.1 U/L [SD 215.7]). Patients in the switch group showed an increase in serum CK levels during alg + pbo treatment in PROPEL (mean CFBL + 57.0 U/L [SD 122.7]), followed by a decrease after switching to cipa + mig in the OLE (mean CFBL −161.0 U/L [SD 269.5]); by week 104, the mean CFBL was similar to that of patients who had received cipa + mig throughout the study (Fig. 3a). Changes from baseline in urine Hex4 levels followed a similar pattern (Fig. 3b).

ERT-experienced patients who were treated with cipa + mig in PROPEL showed an increase in PROMIS–Physical Function SF20a score (an increased score suggests improvement in this measure) and a mean decrease in PROMIS–Fatigue SF8a score (a decrease suggests improvement in this measure) to week 52 (mean CFBL + 2.1 [SD 7.1] and −2.2 [SD 5.9], respectively). Mean CFBL scores remained similar through week 104 (mean CFBL + 1.9 [SD 9.1] and −2.1 [SD 5.6] respectively; Supplementary Fig. S3). Patients who received alg + pbo in PROPEL were generally stable in both PROMIS scores to week 52 but then experienced a change from baseline suggestive of clinical worsening by week 104 (PROMIS–Physical Function score mean CFBL −2.0 [SD 10.2]; PROMIS–Fatigue score mean CFBL + 1.1 [SD 6.9]).

### Efficacy outcomes: ERT-naïve patient cohort

**Fig. 4:**
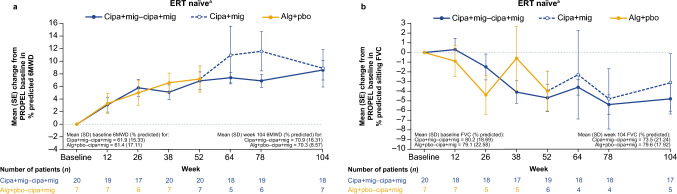
Change from baseline in **a** 6MWD (% predicted) and **b** sitting FVC (% predicted) in ERT-naïve patients. ^a^OLE-ES population excluding outlier. *6MWD* 6-min walk distance; *alg* + *pbo* alglucosidase alfa + placebo; *cipa* + *mig* cipaglucosidase alfa + miglustat; *ERT* enzyme replacement therapy; *FVC* forced vital capacity; *OLE-ES* open-label extension enrolled subjects; *SD* standard deviation; *SE* standard error

ERT-naïve patients in both treatment groups showed numerical increases in % predicted 6MWD in PROPEL to week 52 (mean CFBL + 6.9 [SD 8.2] for the cipa + mig group and + 7.2 [SD 4.5] for the switch group), which were maintained to similar levels during cipa + mig treatment in the OLE to week 104 (mean CFBL + 8.6 [SD 8.6] and + 8.9 [SD 11.7], respectively; Fig. 4a). Absolute 6MWD data in meters are summarized in Supplementary Table S1.

ERT-naïve patients in the cipa + mig group showed a decreased mean GSGC total score (lower scores being suggestive of disease improvement) from baseline to week 52 and scores remained stable at week 104. For patients in the switch group, mean GSGC total score increased (higher scores being suggestive of disease progression) during PROPEL to week 52 but decreased after switching to cipa + mig in the OLE to week 104 (Supplementary Fig. S4).

The cipa + mig group showed a numerical increase in MMT lower extremity scores through week 52 of PROPEL (mean CFBL + 1.5 [SD 2.5]), which increased further through week 104 (mean CFBL + 2.5 [SD 2.7]). In contrast, there was some visit-to-visit variability in the switch group, but scores remained generally stable from baseline to week 104 (mean CFBL + 0.1 [SD 3.1]; Supplementary Fig. S5).

For % predicted FVC, ERT-naïve patients in both treatment groups experienced a decline in PROPEL to week 52 (mean CFBL −4.7 [SD 6.2] for the cipa + mig group and −4.0 [SD 5.1] for the switch group) and stabilization in the OLE with no further decline from week 52 to week 104 (mean CFBL −4.8 [SD 6.5] and −3.1 [SD 6.7] respectively; Fig. 4b).

**Fig. 5:**
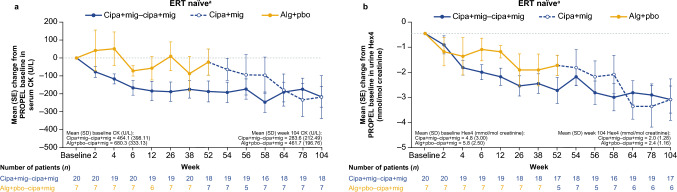
Change from baseline in **a** serum CK and **b** urine Hex4 levels in ERT-naïve patients. ^a^OLE-ES population excluding outlier. *Alg* + *pbo* alglucosidase alfa + placebo; *cipa* + *mig* cipaglucosidase alfa + miglustat; *CK* creatine kinase; *ERT* enzyme replacement therapy; *Hex4* hexose tetrasaccharide; *OLE-ES* open-label extension enrolled subjects; *SD* standard deviation; *SE* standard error

Serum CK and urine Hex4 levels decreased from baseline to week 52 (mean CFBL −187.2 U/L [SD 247.9] for CK and −2.5 mmol/mol [SD 2.3] for Hex4) and remained stable throughout the OLE to week 104 in the cipa + mig group (mean CFBL −216.9 U/L [SD 243.7] and −2.9 mmol/mol [SD 2.5], respectively; Fig. 5a, b). Patients in the switch group showed relative stability in serum CK levels during alg + pbo treatment in PROPEL (mean CFBL −23.1 U/L [SD 193.8]), followed by a decrease to week 104 after switching to cipa + mig in the OLE (mean CFBL −218.6 U/L [SD 316.5]; Fig. 5a). There was a numerical decrease in mean Hex4 levels with alg + pbo treatment from baseline to week 52 in PROPEL (mean CFBL −1.4 mmol/mol [SD 1.0]) that continued through the OLE to week 104 (mean CFBL −2.9 mmol/mol [SD 2.2]; Fig. 5b).

ERT-naïve patients in both treatment groups showed increases in PROMIS–Physical Function SF20a scores (higher scores being suggestive of disease improvement) to week 52 (mean CFBL + 2.5 [SD 8.6] for the cipa + mig group and + 5.1 [SD 7.8] for the switch group) which were maintained at similar levels to week 104 (mean CFBL + 3.6 [SD 9.4] and + 5.4 [SD 10.2], respectively; Supplementary Fig. S6). For PROMIS–Fatigue SF8a score (lower scores being suggestive of disease improvement), ERT-naïve patients had decreased scores relative to baseline at week 52 in PROPEL in both treatment groups (mean CFBL −2.5 [SD 5.6] for the cipa + mig group and −7.7 [SD 8.8] for the switch group). They were generally stable through the OLE to week 104, except the scores returned to near baseline for the week 104 visit in the cipa + mig group (mean CFBL −0.1 [SD 6.7] and −4.6 [SD 10.4] for the switch group; Supplementary Fig. S6).

### Safety

At the time of the data cutoff, 91.8% of patients in the cipa + mig group had received > 24 months of cipa + mig treatment with a maximum treatment duration of 35 months, and 91.9% of patients in the switch group had received > 12 months of cipa + mig treatment (maximum treatment duration 24 months).

A summary of TEAEs for the OLE safety population is shown in Table 2. Overall, a total of 84 (98.8%) patients in the cipa + mig group (throughout PROPEL and the OLE) and 36 (97.3%) in the switch group (during the OLE) experienced at least one TEAE. The most reported TEAEs in both treatment groups were fall (*n* = 35 [41.2%] in the cipa + mig group; *n* = 13 [35.1%] in the switch group), headache (*n* = 30 [35.3%]; *n* = 11 [29.7%], respectively) and arthralgia (*n* = 27 [31.8%]; *n* = 10 [27.0%], respectively; Supplementary Table S2). Thirty-seven patients (43.5%) in the cipa + mig group experienced TEAEs that were deemed by the investigator to be related to either cipaglucosidase alfa or miglustat treatment (or both); 15 patients (40.5%) in the switch group experienced treatment-related TEAEs. The most common treatment-related TEAEs were headache (*n* = 11 [12.9%] in the cipa + mig group and *n* = 4 [10.8%] in the switch group), diarrhea (*n* = 7 [8.2%]; *n* = 2 [5.4%], respectively) and pyrexia (*n* = 6 [7.1%]; *n* = 1 [2.7%], respectively; Table 2). Fourteen patients (16.5%) in the cipa + mig group had a treatment-emergent serious adverse event (TESAE), of which 1 was deemed to be treatment related (anaphylactoid reaction). In the switch group, six patients (16.2%) had a TESAE, of which two were deemed treatment related (urticaria and hypotension; anaphylaxis). Three patients (3.5%) in the cipa + mig group and 2 (5.4%) in the switch group discontinued treatment in PROPEL or the OLE, and these were all due to treatment-related TEAEs (anaphylactoid reaction, urticaria, chills [cipa + mig group]; urticaria and hypotension, anaphylaxis [switch group]).

**Table 2:** Overall summary of TEAEs (safety population)

Patients, *n* (%)	Cipa + mig group (*N* = 85)^a^	Switch group (*N* = 37)^b^	Total patients treated with cipa + mig (*N* = 122)
TEAE	84 (98.8)	36 (97.3)	120 (98.4)
TEAE leading to study drug discontinuation	3 (3.5)	2 (5.4)	5 (4.1)
Treatment-related TEAE	37 (43.5)	15 (40.5)	52 (42.6)
Treatment-related TEAE leading to study drug discontinuation	3 (3.5)^c^	2 (5.4)^d^	5 (4.1)
TESAE	14 (16.5)	6 (16.2)	20 (16.4)
TESAE leading to study drug discontinuation	1 (1.2)	2 (5.4)	3 (2.5)
Treatment-related TESAE	1 (1.2)	2 (5.4)	3 (2.5)
Treatment-related TESAE leading to study drug discontinuation	1 (1.2)	2 (5.4)	3 (2.5)
TESAE leading to death	0	0	0
IAR	27 (31.8)	10 (27.0)	37 (30.3)
Treatment-related TEAE by preferred term occurring in ≥ 2 patients, *n* (%)			
Headache	11 (12.9)	4 (10.8)	15 (12.3)
Diarrhea	7 (8.2)	2 (5.4)	9 (7.4)
Pyrexia	6 (7.1)	1 (2.7)	7 (5.7)
Fatigue	5 (5.9)	2 (5.4)	7 (5.7)
Nausea	5 (5.9)	2 (5.4)	7 (5.7)
Dizziness	4 (4.7)	0 (0.0)	4 (3.3)
Pruritus	3 (3.5)	1 (2.7)	4 (3.3)
Urticaria	2 (2.4)	3 (8.1)	5 (4.1)
Somnolence	1 (1.2)	2 (5.4)	3 (2.5)
Abdominal pain upper	2 (2.4)	1 (2.7)	3 (2.5)
Abdominal distension	3 (3.5)	0 (0.0)	3 (2.5)
Abdominal pain	0 (0.0)	2 (5.4)	2 (1.6)

A TEAE was defined as any adverse event that started on or after the first dose of study drug. Any AE that occurred after 30 days from last dose of study drug in PROPEL and before the first dose of study drug in the OLE was not counted as treatment emergent. A treatment-related TEAE was defined as TEAE with a definite, probable, or possible relationship to study drug as judged by the investigator

*AE* adverse event; *cipa* + *mig* cipaglucosidase alfa + miglustat; *IAR* infusion-associated reaction; *TEAE* treatment-emergent adverse event; *TESAE* treatment-emergent serious adverse event

^a^Includes data from patients treated with cipa + mig in PROPEL who may or may not have continued cipa + mig in the OLE, including data from both PROPEL and the OLE

^b^Includes data from the OLE only

^c^Two patients discontinued treatment during PROPEL due to anaphylactoid reaction and chills, respectively, and one patient discontinued treatment during the OLE due to urticaria

^d^Two patients discontinued from the OLE due to urticaria and hypotension, and anaphylaxis, respectively

Although most TEAEs were mild or moderate in severity, 13 patients (15.3%) in the cipa + mig group experienced a total of 19 severe TEAEs (abdominal pain, chills, anaphylactoid reaction, COVID-19 pneumonia, accidental overdose, ankle fracture, fall, femur fracture, fibula fracture, hip fracture, tibia fracture, irregular heartbeat, arthralgia, intervertebral disc protrusion, dyspnea, pruritus, urticaria, aortic aneurysm, and flushing). Four severe TEAEs (anaphylactoid reaction, urticaria, pruritus, and chills) were considered IARs and related to treatment. Nine severe TEAEs (arrhythmia, pancreatitis, fatigue, pain, bile duct stone, alanine transaminase [ALT] increased, aspartate transaminase [AST] increased, joint swelling, and weight-bearing difficulty) were experienced by three patients (8.1%) in the switch group, none of which were deemed to be IARs or related to the study drug treatment.

Overall, no clinically meaningful changes in vital signs, clinical safety laboratory assessments, physical exams or electrocardiograms were observed. No deaths occurred during the study.

#### Immunogenicity

For the evaluation of the immunogenicity of cipa + mig, all available immunogenicity samples were analyzed through the data cutoff date, and data were assessed by treatment groups and in subgroups by ERT status (experienced or naïve) at the start of PROPEL. Beyond week 104 (week 52 of the OLE), immunogenicity results were not available for all patients, and the proportions presented are based on the number of patients with samples available for analysis. At the start of the OLE, most patients (78.6–100%) had positive specific anti-rhGAA antibodies regardless of their PROPEL treatment group or ERT experience prior to PROPEL. The proportion of patients with antibodies remained stable from baseline across the OLE study visits (86.2–100% by the last study visit). Most patients had positive specific antibodies with detectable titers (defined as ≥ 100) at the start of the OLE, and the proportion of patients with these antibodies remained high and stable from baseline across study visits.

The proportions of patients with treatment-induced, treatment-boosted and treatment-emergent antibodies ranged from 0% to 14%, 5.3% to 50% and 15.8% to 53.6%, respectively, across all four subgroups (ERT-experienced and ERT-naïve patients in each treatment group). The proportions of patients positive for at least one type of NAb at the start of the OLE was 25–35.7% across all subgroups, and ranged from 53.6% to 63.2% across all subgroups until week 104 or week 130 (week 52 or week 78 of the OLE). The proportion of patients with positive specific antibodies cross reactive to alglucosidase alfa across all subgroups was 18.2–100% at the start of the OLE and 43.9–100% until week 104 or week 130 (week 52 or week 78 of the OLE).

Patient-level analyses of the association between immunogenicity endpoints (total, cross-reactive, and NAbs) and safety were undertaken (Supplementary information). Overall, the weight of evidence does not support an association.

## Discussion

LOPD is a progressive disease [1], and if left untreated, many patients will require wheelchairs and ventilatory support as skeletal muscle function and strength decline [7, 9]. Life expectancy is greatly reduced, with respiratory failure being the leading cause of morbidity and mortality for untreated patients with LOPD [7, 9, 29, 30]. The development of ERT was a major advance in the treatment of patients with LOPD [31]. However, several long-term studies with the standard-of-care ERT alglucosidase alfa have shown that, after initial improvements, many patients experience a decline in multiple outcome measures after 3–5 years of treatment [15–18], highlighting the need for better treatments with long-term effectiveness [32].

The phase III, double-blind, randomized PROPEL study was the first head-to-head study in patients with LOPD previously treated with alglucosidase alfa (ERT experienced) or who were treatment naïve, and is the largest study of ERT for Pompe disease to date [25]. Results from PROPEL demonstrated clinically meaningful improvements in motor and respiratory functions at week 52 in patients treated with cipa + mig compared with those treated with alg + pbo. This OLE was conducted to assess the efficacy and safety of cipa + mig in the longer term (2 years of treatment in those continuing cipa + mig treatment) and in switched patients initially randomized to alg + pbo. Results up to week 52 of the OLE (104 weeks from the PROPEL baseline) show that any improvements gained in motor and respiratory function, biomarker levels and PROs during PROPEL were maintained in those continuing treatment with cipa + mig, regardless of previous ERT status (experienced or naïve). This is consistent with data from the smaller, open-label phase I/II study ATB200-02, in which ambulatory patients with LOPD showed long-term stability in 6MWD and FVC with cipa + mig over a treatment period of up to 48 months [33, 34]. Importantly, stability in key efficacy measures such as 6MWD can be considered a beneficial outcome in the context of a progressive disease like LOPD [16, 19].

In PROPEL, improvements in the primary endpoint, 6MWD, at week 52 numerically favored cipa + mig versus alglucosidase alfa, but the difference did not reach statistical significance. The key secondary endpoint, % predicted FVC, showed a nominally significant benefit for cipa + mig versus alglucosidase alfa [25]. Importantly, 77% of patients in PROPEL had previously received ERT with alglucosidase alfa for a mean duration of > 7 years [25]. For these ERT-experienced patients, 6MWD and FVC nominally significantly improved with cipa + mig versus alglucosidase alfa at week 52 of PROPEL, whereas nominal significance was not reached for either endpoint in ERT-naïve patients [25]. Continuing treatment with cipa + mig from PROPEL or switching from alglucosidase alfa to cipa + mig led to small improvements or stability in the key functional outcomes 6MWD and FVC over the first year of the OLE for both ERT-experienced and ERT-naïve patients.

Notably, in our study, ERT-experienced patients in the switch group (i.e. those who were switched from alglucosidase alfa to cipa + mig at the start of the OLE) did not follow the same trajectory for 6MWD as ERT-experienced patients who were randomized to cipa + mig at the start of PROPEL (i.e. 52 weeks earlier). Patients randomized to cipa-mig at the start of PROPEL showed a potentially clinically meaningful increase in 6MWD at week 52 [25], while patients who were switched at the start of the OLE showed stability from week 52 to week 104. The exact reasons for this are unclear; however, study fatigue, switching from a blinded to an open-label study and the relatively low number of patients in the switch group may have contributed to this result.

Serum CK and urine Hex4 are biomarkers of muscle damage and skeletal glycogen clearance, respectively. Both are elevated in patients with Pompe disease [35, 36]. Although they have not been clinically validated as surrogate measures of treatment efficacy in LOPD, correlations between levels of CK and Hex4 and clinical outcomes have been seen in a population of pediatric LOPD and IOPD patients. In addition, improvements in levels of these biomarkers were seen with higher doses of ERT [37]. A clear difference between the cipa + mig and the switch groups was seen for the assessments of serum CK and urine Hex4. Both biomarkers were markedly reduced in the cipa + mig group versus the alglucosidase alfa group in PROPEL [25]. After switching to cipa + mig in the OLE, ERT-naïve and ERT-experienced patients in the switch group showed a rapid decrease in biomarker levels, reaching similar levels to the cipa + mig group by week 104.

PROs (PROMIS–Physical Function and PROMIS–Fatigue) showed a similar trend to the functional efficacy outcomes over the OLE, with scores remaining stable over the first year of the OLE. While there was a notable visit-to-visit variability in the small subgroup of ERT-naïve patients, no trend toward a deterioration of patient-reported physical function or fatigue was seen. Although PROMIS measures are not specific to patients with Pompe disease, patients have rated several PROMIS scales, including Physical Function and Fatigue, as important to representing the impact on their health-related quality of life [38].

No new safety signals were identified in patients continuing cipa + mig from PROPEL or in the switch group. Cipa + mig was generally well tolerated in patients treated for up to 35 months, and an increased frequency of treatment-related TEAEs was not observed with an increased duration of cipa + mig exposure. The most frequently reported TEAEs in the OLE were consistent with the current safety profile of cipa + mig [25, 34]. The incidence of IARs with cipa + mig in the OLE was consistent with that of IARs with cipa + mig in PROPEL [25]. The tolerability of cipa + mig was further supported by the low number of patients who discontinued treatment during the OLE due to adverse events (*n* = 3); 90.7% of all patients who received treatment in the OLE were still ongoing in the study at the data cutoff.

ERT for Pompe disease relies on the effective delivery of active rhGAA to the lysosome of muscle cells. Cipaglucosidase alfa is a novel rhGAA with a high amount of CHO-cell derived bis-M6P glycans and enhanced glycosylation to ensure processing of the enzyme into a mature form with maximum catalytic activity. Combination with the enzyme stabilizer miglustat enhances the half-life of the enzyme in the blood to improve biodistribution and helps maintain catalytic activity prior to uptake into the muscle, where cipaglucosidase alfa dissociates from miglustat [19–21]. The results of PROPEL and the OLE support the clinical effectiveness of cipa + mig. Acknowledging the inherent individual variability in these biomarkers, the group-level improvement, shown as mean change from the PROPEL baseline, in levels of Hex4 and CK in patients who switched to cipa + mig, suggest increased glycogen clearance and reduced muscle cell damage, which may be due to enhanced uptake and activity of the enzyme within muscle cells leading to reduced glycogen storage. The general improvement and/or stability of functional outcomes in patients treated with cipa + mig throughout both studies and in those who switched to cipa + mig in the OLE demonstrate that the enhanced activity of the enzyme at a cellular level translates to beneficial outcomes for patients.

### Limitations

The OLE was unblinded and data were analyzed descriptively, without statistical comparisons. In addition, as LOPD is a rare disease, the sample size was relatively small, particularly in the subgroup of ERT-naïve patients. The heterogeneous nature of LOPD, spanning a wide spectrum of manifestations, disease severity, progression rates and treatment responses, may have introduced variability into the small dataset. However, the inter-patient variability noted in our study was similar to that seen in other recent studies in LOPD [34, 39]. At the current data-cut, patients received treatment with cipa + mig for up to 2 years; further, longer-term data will be collected.

## Conclusions

Data from the OLE of the PROPEL study show that patients with LOPD who were treated with cipa + mig for 2 years (104 weeks) maintained improvements relative to baseline in motor function, muscle strength and biomarker levels, regardless of whether they were treatment naïve or had been treated with alglucosidase alfa prior to PROPEL. Respiratory function and PROs were stable throughout the OLE in these patients. Patients who were switched from alglucosidase alfa to cipa + mig at the start of the OLE demonstrated stability in motor and respiratory function, muscle strength, and PROs over the first year of cipa + mig treatment. Biomarker levels improved during the OLE in all patients who switched to cipa + mig in the OLE. Cipa + mig was well tolerated and no new safety signals were identified. Overall, data support the long-term benefits of cipa + mig treatment for patients with LOPD.

### Supplementary Information

Below is the link to the electronic supplementary material.

Supplementary file1 (PDF 542 KB)

Supplementary file2 (PDF 1757 KB)

## Data Availability

Data sharing proposals and requests will be reviewed on a case-by-case basis. Requests for data should be addressed to Mitchell Goldman at mgoldman@amicusrx.com. Requests will be reviewed by a medical steering committee.

## References

[1] Cabello J, Marsden D (2017). Pompe disease: clinical perspectives. Orphan Drugs Res Rev.

[2] Park KS (2021). Carrier frequency and predicted genetic prevalence of Pompe disease based on a general population database. Mol Genet Metab Rep.

[3] Nino MY, In’t Groen SLM, Bergsma AJ (2019). Extension of the Pompe mutation database by linking disease-associated variants to clinical severity. Hum Mutat.

[4] Raben N, Wong A, Ralston E (2012). Autophagy and mitochondria in Pompe disease: nothing is so new as what has long been forgotten. Am J Med Genet C Semin Med Genet.

[5] Cupler EJ, Berger KI, Leshner RT (2012). Consensus treatment recommendations for late-onset Pompe disease. Muscle Nerve.

[6] Kishnani PS, Hwu WL, Mandel H (2006). A retrospective, multinational, multicenter study on the natural history of infantile-onset Pompe disease. J Pediatr.

[7] Kishnani PS, Howell RR (2004). Pompe disease in infants and children. J Pediatr.

[8] van der Ploeg AT, Reuser AJ (2008). Pompe's disease. Lancet.

[9] Kishnani PS, Steiner RD, Bali D (2006). Pompe disease diagnosis and management guideline. Genet Med.

[10] Toscano A, Rodolico C, Musumeci O (2019). Multisystem late onset Pompe disease (LOPD): an update on clinical aspects. Ann Transl Med.

[11] Tarnopolsky M, Katzberg H, Petrof BJ (2016). Pompe disease: diagnosis and management. Evidence-based guidelines from a Canadian expert panel. Can J Neurol Sci.

[12] van der Ploeg AT, Kruijshaar ME, Toscano A (2017). European consensus for starting and stopping enzyme replacement therapy in adult patients with Pompe disease: a 10-year experience. Eur J Neurol.

[13] European Medicines Agency (2014) Committee for medicinal products for human use (CHMP) opinion on Myozyme. Available from https://www.ema.europa.eu/en/medicines/human/EPAR/myozyme. Accessed 31 Jan 2024

[14] van der Ploeg AT, Clemens PR, Corzo D (2010). A randomized study of alglucosidase alfa in late-onset Pompe's disease. N Engl J Med.

[15] Gutschmidt K, Musumeci O, Diaz-Manera J (2021). STIG study: real-world data of long-term outcomes of adults with Pompe disease under enzyme replacement therapy with alglucosidase alfa. J Neurol.

[16] Harlaar L, Hogrel JY, Perniconi B (2019). Large variation in effects during 10 years of enzyme therapy in adults with Pompe disease. Neurology.

[17] Schoser B, Stewart A, Kanters S (2017). Survival and long-term outcomes in late-onset Pompe disease following alglucosidase alfa treatment: a systematic review and meta-analysis. J Neurol.

[18] Semplicini C, De Antonio M, Taouagh N (2020). Long-term benefit of enzyme replacement therapy with alglucosidase alfa in adults with Pompe disease: prospective analysis from the French Pompe Registry. J Inherit Metab Dis.

[19] Do HV, Khanna R, Gotschall R (2019). Challenges in treating Pompe disease: an industry perspective. Ann Transl Med.

[20] Xu S, Lun Y, Frascella M (2019). Improved efficacy of a next-generation ERT in murine Pompe disease. JCI Insight.

[21] Selvan N, Mehta N, Venkateswaran S (2021). Endolysosomal N-glycan processing is critical to attain the most active form of the enzyme acid alpha-glucosidase. J Biol Chem.

[22] Johnson FK, Kang J, Mondick J et al (2022) Mechanism of action, plasma total GAA protein profiles and PK/PD relationships differ between cipaglucosidase alfa/miglustat and alglucosidase alfa in patients with late-onset Pompe disease. In: World symposium, San Diego, CA, USA

[23] Blair HA (2023). Cipaglucosidase alfa: first approval. Drugs.

[24] Meena NK, Ralston E, Raben N (2020). Enzyme replacement therapy can reverse pathogenic cascade in Pompe disease. Mol Ther Methods Clin Dev.

[25] Schoser B, Roberts M, Byrne BJ (2021). Safety and efficacy of cipaglucosidase alfa plus miglustat versus alglucosidase alfa plus placebo in late-onset Pompe disease (PROPEL): an international, randomised, double-blind, parallel-group, phase 3 trial. Lancet Neurol.

[26] Byrne BJ, Kishnani PS, Case LE (2011). Pompe disease: design, methodology, and early findings from the Pompe Registry. Mol Genet Metab.

[27] Enright PL, Sherrill DL (1998). Reference equations for the six-minute walk in healthy adults. Am J Respir Crit Care Med.

[28] Quanjer PH, Stanojevic S, Cole TJ (2012). Multi-ethnic reference values for spirometry for the 3–95-yr age range: the global lung function 2012 equations. Eur Respir J.

[29] Gungor D, de Vries JM, Hop WC (2011). Survival and associated factors in 268 adults with Pompe disease prior to treatment with enzyme replacement therapy. Orphanet J Rare Dis.

[30] Winkel LP, Hagemans ML, van Doorn PA (2005). The natural course of non-classic Pompe's disease; a review of 225 published cases. J Neurol.

[31] Meena NK, Raben N (2020). Pompe disease: new developments in an old lysosomal storage disorder. Biomolecules.

[32] Puertollano R, Raben N (2021). New therapies for Pompe disease: are we closer to a cure?. Lancet Neurol.

[33] National Library of Medicine (2023) First-in-human study to evaluate safety, tolerability, and PK of intravenous ATB200 alone and when co-administered with oral AT2221. 2023 [cited 2023 24 Feb]. Available from https://clinicaltrials.gov/ct2/show/NCT02675465. Accessed 31 Jan 2024

[34] Byrne BJ, Schoser B, Kishnani PS (2023). Long-term safety and efficacy of cipaglucosidase alfa plus miglustat in individuals living with Pompe disease: an open-label phase I/II study (ATB200-02). J Neurol.

[35] An Y, Young SP, Kishnani PS (2005). Glucose tetrasaccharide as a biomarker for monitoring the therapeutic response to enzyme replacement therapy for Pompe disease. Mol Genet Metab.

[36] Brancaccio P, Lippi G, Maffulli N (2010). Biochemical markers of muscular damage. Clin Chem Lab Med.

[37] Khan AA, Case LE, Herbert M (2020). Higher dosing of alglucosidase alfa improves outcomes in children with Pompe disease: a clinical study and review of the literature. Genet Med.

[38] Harfouche M, Kishnani PS, Krusinska E (2020). Use of the patient-reported outcomes measurement information system (PROMIS(R)) to assess late-onset Pompe disease severity. J Patient Rep Outcomes.

[39] Kishnani PS, Díaz-Manera J, Kushlaf H (2023). Efficacy and safety of avalglucosidase alfa in participants with late-onset Pompe disease after 145 weeks of treatment during the COMET trial. Mol Gen Metab.

