# Supplementary material for: 104-week efficacy and safety of cipaglucosidase alfa plus miglustat in adults with late-onset Pompe disease: a phase III open-label extension study (ATB200-07)
Source: J Neurol. 2024 Feb 28;271(5):2810–23. doi: 10.1007/s00415-024-12236-0 (PMC11055775; doi:10.1007/s00415-024-12236-0)
Supplement: Supplementary file 1 — Supplementary file1 (PDF 542 KB) [file 415_2024_12236_MOESM1_ESM.pdf]

Supplementary material

104-week efficacy and safety of cipaglucosidase alfa plus miglustat in adults with late-onset Pompe disease: a phase III open-label extension study (ATB200-07)

Benedikt Schoser,[1] Priya S. Kishnani,[2] Drago Bratkovic,[3] Barry J. Byrne,[4] Kristl G. Claeys,[5] Jordi Díaz-Manera,[6] Pascal Laforêt,[7] Mark Roberts,[8] Antonio Toscano,[9] Ans T. van der Ploeg,[10] Jeff Castelli,[11] Mitchell Goldman,[11] Fred Holdbrook,[11] Sheela Sitaraman Das,[11] Yasmine Wasfi,[11] Tahseen Mozaffar,[12] on behalf of the ATB200-07 study group

Journal title: *Journal of Neurology*

Corresponding author: Prof Dr med Benedikt Schoser; Friedrich-Baur-Institute at the Department of Neurology, LMU University Hospital, LMU Munich, Munich, Germany; benedikt.schoser@med.uni-muenchen.de

Relationship between immunogenicity and safety in PROPEL and the OLE

The association between immunogenicity endpoints and safety (adverse events [AEs], stratified by system organ class and infusion-associated reactions [IARs]), was evaluated by patient level analysis. The analyses were limited by the small numbers of patients who were negative for total anti-drug antibodies (ADA) and/or the small numbers of patients with AEs.

Relationship between AEs or IARs and ADAs or neutralizing antibodies (NAbs)

The mean number of AEs per patient with or without ADA or NAbs at baseline or post-treatment (between-patients), and the AE per month rates before and after the onset of ADA or NAbs (within-patients) were compared. None of these comparisons yielded statistically significant results, suggesting no apparent effects of these immunogenicity endpoints on the number of AEs per patient or the AE rates before versus after the patients became ADA positive.

Similarly, the mean number of IARs per patient with or without ADA or NAbs at baseline or post-treatment (between-patients), and the IARs per month rates before and after the onset of ADA or NAbs (within-patients) were compared. Certain between-patient analyses showed an association, but some conclusions were limited by the small numbers of patients who were negative for total ADA.

The majority of between-patient and within-patient analyses showed no or inconsistent effects. Overall, the weight of evidence does not support an association between these immunogenicity endpoints and the number of IARs per patient or the IAR rates before versus after the patients became ADA positive. Descriptive analyses of the relationship between ADA, including anti-recombinant human acid α-glucosidase (rhGAA) immunoglobulin E (IgE), and IARs also showed that there was no clear trend in IAR occurrence with the incidence of anti-rhGAA IgE or with total ADA titers.

Relationship between IAR-TEAEs leading to study drug discontinuation and anti-rhGAA antibodies

A total of five patients treated with cipa+mig experienced treatment-emergent AEs that were IARs leading to study drug discontinuation. All five patients had positive specific anti-rhGAA antibodies. Of these five patients, three discontinued after a serious IAR. Of these three patients, two had at least one positive result of anti-rhGAA IgE post-treatment with cipaglucosidase alfa, while one patient remained negative. Both patients who discontinued after a non-serious IAR had at least one positive result of anti rhGAA IgE post-treatment with cipaglucosidase alfa.

Supplementary Table S1 Change in 6MWD (meters) from PROPEL baseline (OLE-ES population excluding outlier)

	ERT experienced				ERT naïve[a]			
	Cipa+mig group		Switch group		Cipa+mig group		Switch group	
	n	**Mean (SD)**	***n***	**Mean (SD)**	***n***	**Mean (SD)**	***n***	**Mean (SD)**
**6MWD, m**								
**Baseline**	62	342.4 (100.40)	29	335.0 (116.03)	20	393.6 (112.39)	7	420.9 (135.75)
**Week 104**	56	367.1 (129.11)	26	330.5 (142.54)	18	428.0 (107.74)	7	469.3 (106.30)
**Change from PROPEL baseline in 6MWD, m**								
**Week 12**	61	10.9 (23.18)	29	5.3 (21.68)	19	17.1 (20.97)	7	20.5 (22.12)
**Week 26**	55	12.9 (30.64)	27	5.6 (26.60)	17	32.2 (36.75)	6	30.3 (17.94)
**Week 38**	58	13.3 (32.82)	28	4.0 (28.90)	20	25.2 (46.25)	7	38.5 (19.46)
**Week 52**	61	16.3 (39.46)	29	0.7 (39.84)	20	33.4 (48.70)	7	38.3 (29.32)
**Week 64**	55	14.2 (43.27)	27	5.7 (49.81)	18	35.5 (47.65)	5	63.3 (49.66)
**Week 78**	56	19.8 (53.17)	26	0.8 (44.88)	19	31.8 (47.83)	6	66.2 (49.47)
**Week 104**	56	14.2 (53.44)	26	−8.8 (46.19)	18	38.8 (51.03)	7	48.3 (70.56)

[a]Excludes outlier. *6MWD* 6-minute walk distance; *cipa+mig* cipaglucosidase alfa+miglustat; *ERT* enzyme replacement therapy; *OLE-ES* open-label extension enrolled subjects; *SD* standard deviation

Supplementary Table S2 Incidence of TEAEs occurring in ≥ 10% patients (in any group) by preferred term (safety population)

**Preferred term**	**Cipa+mig group (*N* = 85)[a]**	**Switch group (*N* = 37)[b]**	**Total patients treated with cipa+mig (*N* = 122)**
Patients with any TEAE	84 (98.8)	36 (97.3)	120 (98.4)
Fall	35 (41.2)	13 (35.1)	48 (39.3)
Headache	30 (35.3)	11 (29.7)	41 (33.6)
Arthralgia	27 (31.8)	10 (27.0)	37 (30.3)
Nasopharyngitis	24 (28.2)	1 (2.7)	25 (20.5)
Myalgia	23 (27.1)	7 (18.9)	30 (24.6)
Back pain	19 (22.4)	5 (13.5)	24 (19.7)
Pain in extremity	17 (20.0)	8 (21.6)	25 (20.5)
Diarrhea	17 (20.0)	3 (8.1)	20 (16.4)
Nausea	16 (18.8)	5 (13.5)	21 (17.2)
Fatigue	15 (17.6)	6 (16.2)	21 (17.2)
Oropharyngeal pain	15 (17.6)	2 (5.4)	17 (13.9)
Musculoskeletal pain	14 (16.5)	3 (8.1)	17 (13.9)
Urinary tract infection	14 (16.5)	3 (8.1)	17 (13.9)
COVID-19	14 (16.5)	3 (8.1)	17 (13.9)
Pyrexia	13 (15.3)	2 (5.4)	15 (12.3)
Muscle spasm	12 (14.1)	2 (5.4)	14 (11.5)
Dizziness	12 (14.1)	2 (5.4)	14 (11.5)
Cough	11 (12.9)	1 (2.7)	12 (9.8)
Vaccination complication	11 (12.9)	4 (10.8)	15 (12.3)
Upper respiratory tract infection	10 (11.8)	3 (8.1)	13 (10.7)
Abdominal pain	8 (9.4)	4 (10.8)	12 (9.8)
Contusion	7 (8.2)	6 (16.2)	13 (10.7)

A TEAE was defined as any event that started on or after the first dose of respective study drug. Any AE that occurred after 30 days from last dose of study drug in PROPEL and before the first dose of study drug in ATB200-07 was not counted as treatment emergent. A patient experiencing the same TEAE multiple times was counted once for the corresponding preferred term. [a]Includes data from patients treated with cipa+mig in PROPEL who may or may not have continued cipa+mig in the OLE, including data from both PROPEL and the OLE; [b]Includes data from the OLE only. *AE* adverse event; *cipa+mig* cipaglucosidase alfa+miglustat; *TEAE* treatment-emergent adverse event

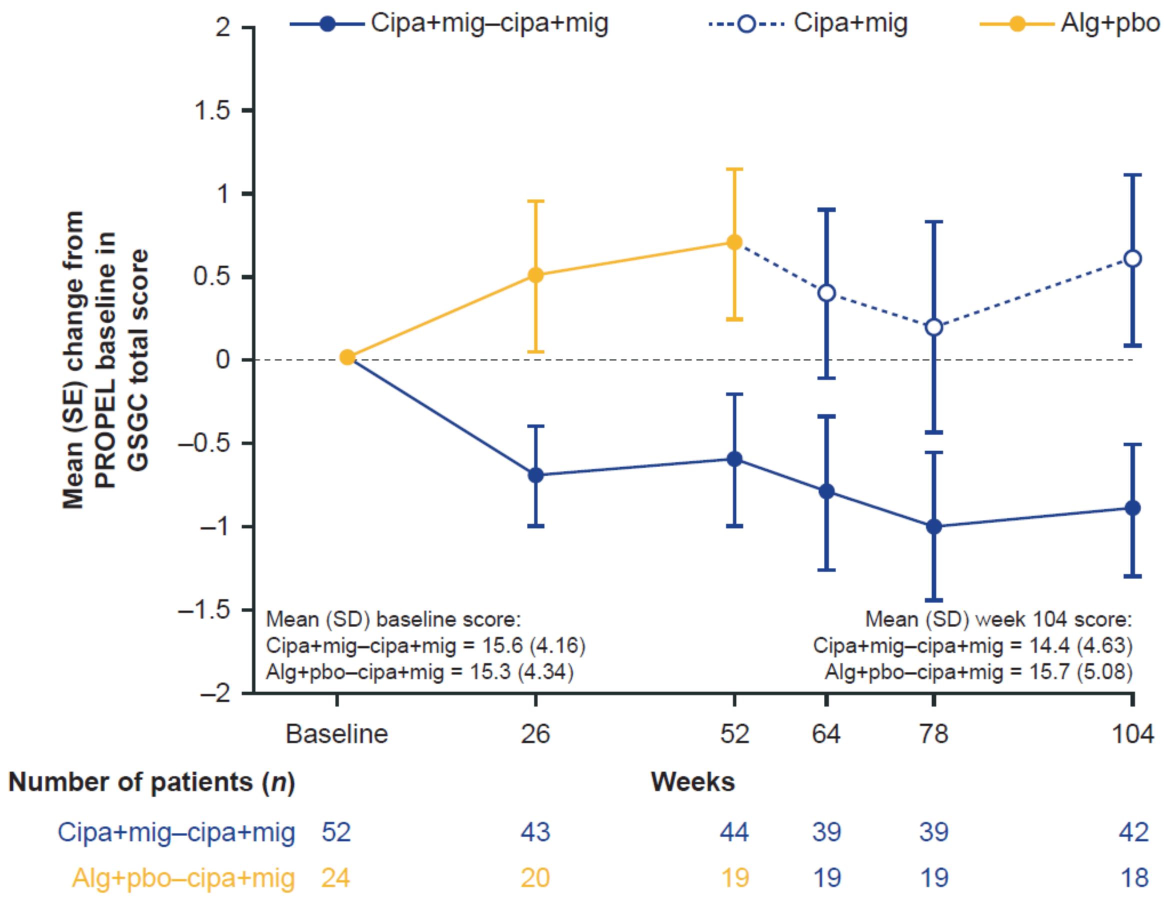

Supplementary Fig. S1 Change from baseline in GSGC total score for ERT-experienced patients (OLE-ES population). *Alg+pbo* alglucosidase alfa+placebo; *cipa+mig* cipaglucosidase alfa+miglustat; *ERT* enzyme replacement therapy; GSGC Gait, Stair, Gowers' maneuver, Chair; *OLE-ES* open-label extension enrolled subjects; *SD* standard deviation; *SE* standard error

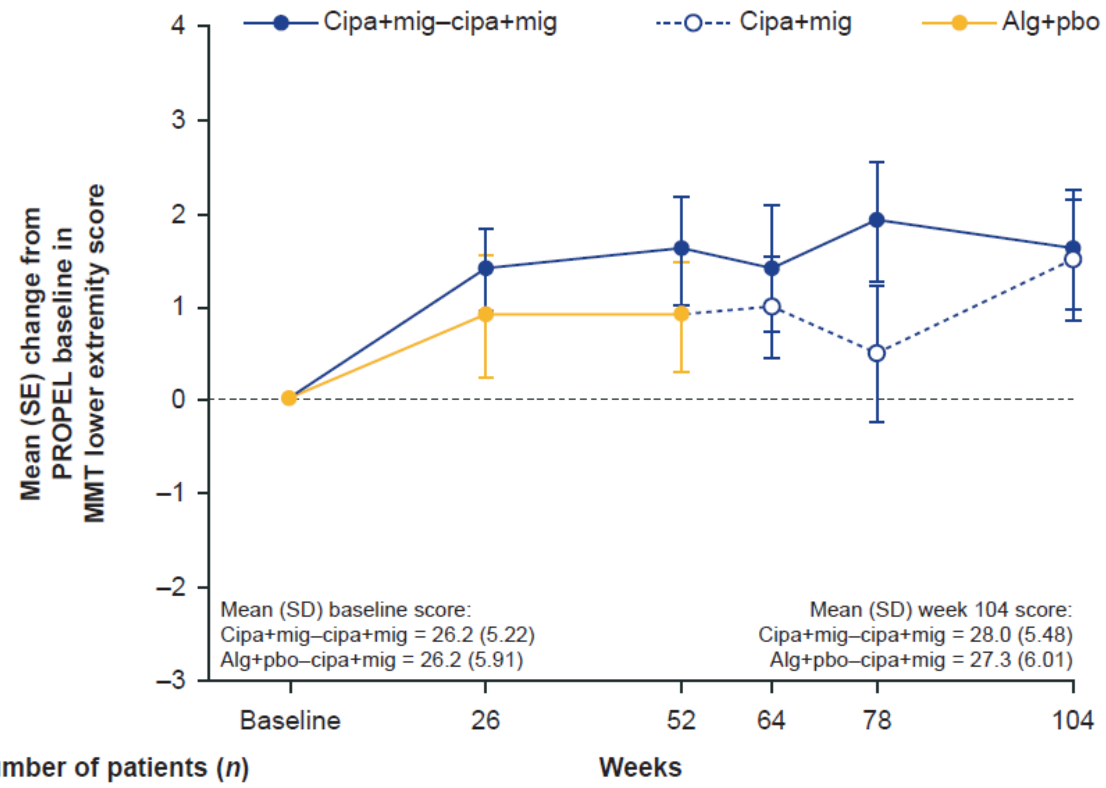

Number of patients (*n*)						
Cipa+mig–cipa+mig	61	50	54	50	50	51
Alg+pbo–cipa+mig	26	23	25	22	22	20

Supplementary Fig. S2 Change from baseline in MMT lower extremity score for ERT-experienced patients (OLE-ES population). *Alg+pbo* alglucosidase alfa+placebo; cipa+mig cipaglucosidase alfa+miglustat; *ERT* enzyme replacement therapy; *MMT* manual muscle testing; *OLE-ES* open-label extension enrolled subjects; *SD* standard deviation; *SE* standard error

a

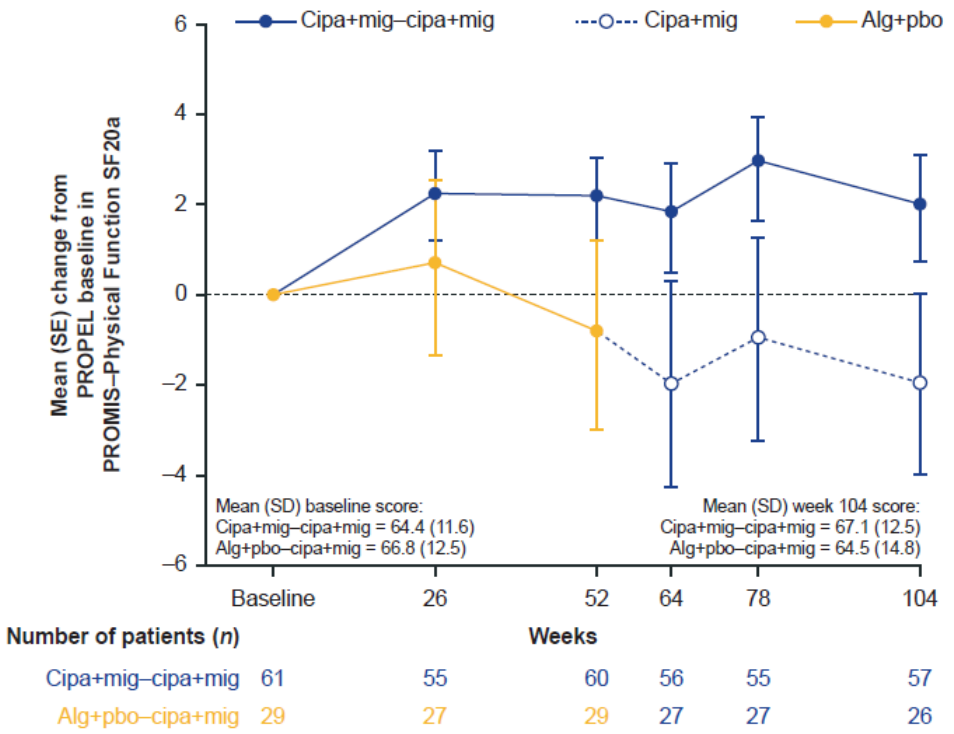

Cipa+mig–cipa+mig
Cipa+mig
Alg+pbo
Mean (SE) change from PROPEL baseline in PROMIS–Physical Function SF20a
6
4
2
0
–2
–4
–6
Mean (SD) baseline score:
Cipa+mig–cipa+mig = 64.4 (11.6)
Alg+pbo–cipa+mig = 66.8 (12.5)
Mean (SD) week 104 score:
Cipa+mig–cipa+mig = 67.1 (12.5)
Alg+pbo–cipa+mig = 64.5 (14.8)
Baseline
26
52
64
78
104
Weeks
Number of patients (n)
Cipa+mig–cipa+mig 61 55 60 56 55 57
Alg+pbo–cipa+mig 29 27 29 27 27 26

b

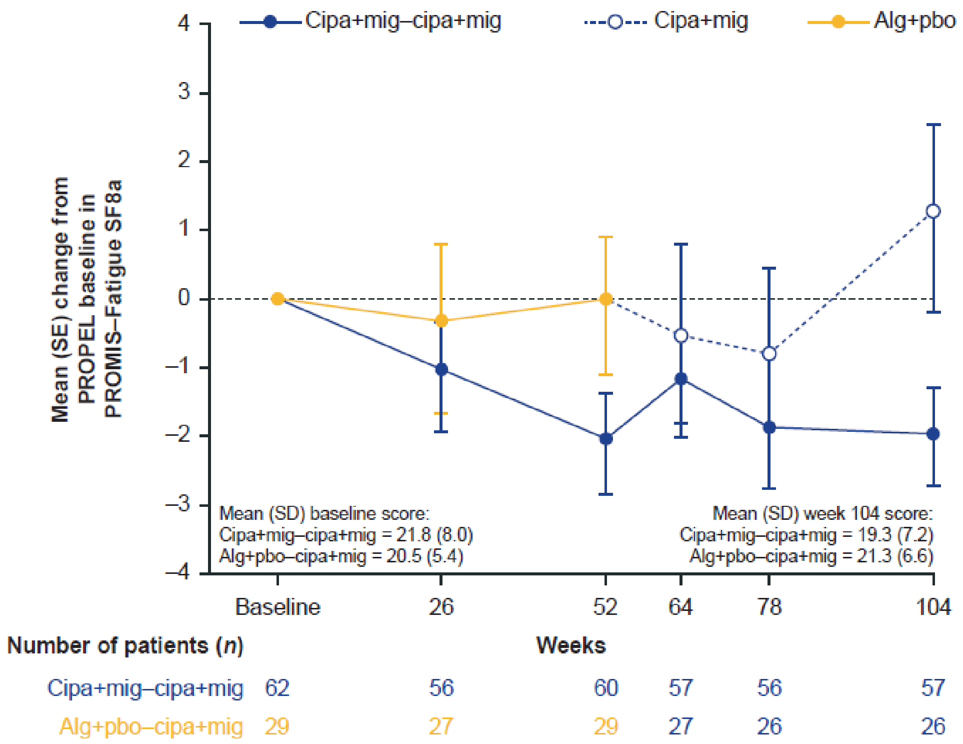

Cipa+mig–cipa+mig
Cipa+mig
Alg+pbo
Mean (SE) change from PROPEL baseline in PROMIS–Fatigue SF8a
4
3
2
1
0
–1
–2
–3
–4
Mean (SD) baseline score:
Cipa+mig–cipa+mig = 21.8 (8.0)
Alg+pbo–cipa+mig = 20.5 (5.4)
Mean (SD) week 104 score:
Cipa+mig–cipa+mig = 19.3 (7.2)
Alg+pbo–cipa+mig = 21.3 (6.6)
Baseline
26
52
64
78
104
Weeks
Number of patients (n)
Cipa+mig–cipa+mig 62 56 60 57 56 57
Alg+pbo–cipa+mig 29 27 29 27 26 26

Supplementary Fig. S3 Change from baseline in PROMIS–Physical Function SF20a (a) and PROMIS–Fatigue score SF8a (b) for ERT-experienced patients (OLE-ES population). *Note:* The total score ranges from 20 to 100 for PROMIS–Physical Function, with higher scores indicating less impact on physical function. The total score is calculated by summing up scores (1–5) across all 20 items. For PROMIS–Fatigue, the total score ranges from 8 to 40, with a lower score indicating less impact by fatigue and is calculated by summing up scores (1–5) across all 8 items. If baseline scores were partially missing (missing only specific items), the missing item scores were imputed with the average of all non-missing values for the specific items (from both treatment groups combined). The item scores (observed and imputed) were then summed up as the baseline total score. If baseline scores were completely missing, baseline total score was imputed with the average of all observed baseline total scores (from both treatment groups combined), provided there was ≥ 1 post-baseline total score. Item scores were not imputed in this case. If post-baseline scores were partially missing but ≥ 50% of items were available, the total score was calculated as the average of non-missing items multiplied by the total number of items expected. If post-baseline scores were completely missing or < 50% of items were available, the total score was not calculated and set to missing.' The baseline is the last non-missing value on or prior to the administration of the first dose of study drug in PROPEL. *Alg+pbo* alglucosidase alfa+placebo; *cipa+mig* cipaglucosidase alfa+miglustat; *ERT* enzyme replacement therapy; *OLE-ES* open-label extension enrolled subjects; *PROMIS* Patient-Reported Outcomes Measurement Information System; *SD* standard deviation; *SE* standard error

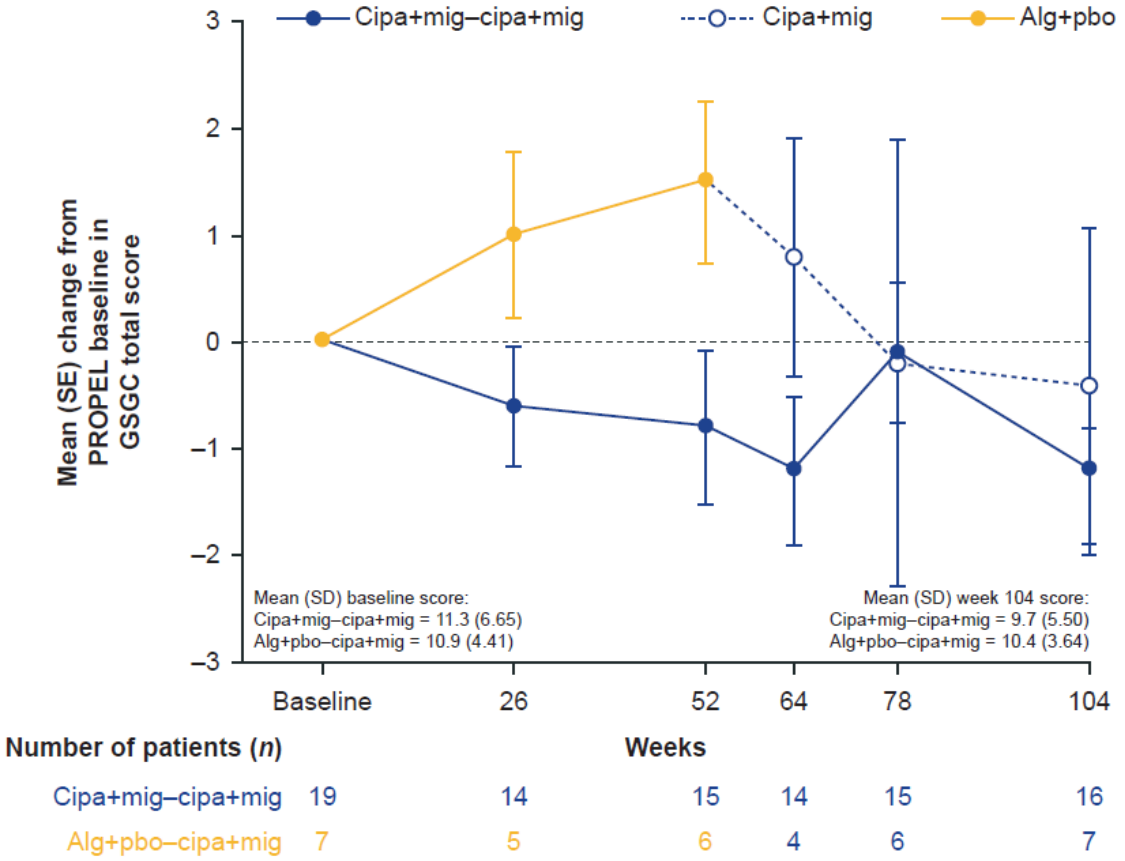

Supplementary Fig. S4 Change from baseline in GSGC total score for ERT-naïve patients (OLE-ES population excluding outlier). *Alg+pbo* alglucosidase alfa+placebo; *cipa+mig* cipaglucosidase alfa+miglustat; *ERT* enzyme replacement therapy; *GSGC* Gait, Stairs, Gowers’ maneuver, Chair; *OLE-ES* open-label extension enrolled subjects; *SD* standard deviation; *SE* standard error

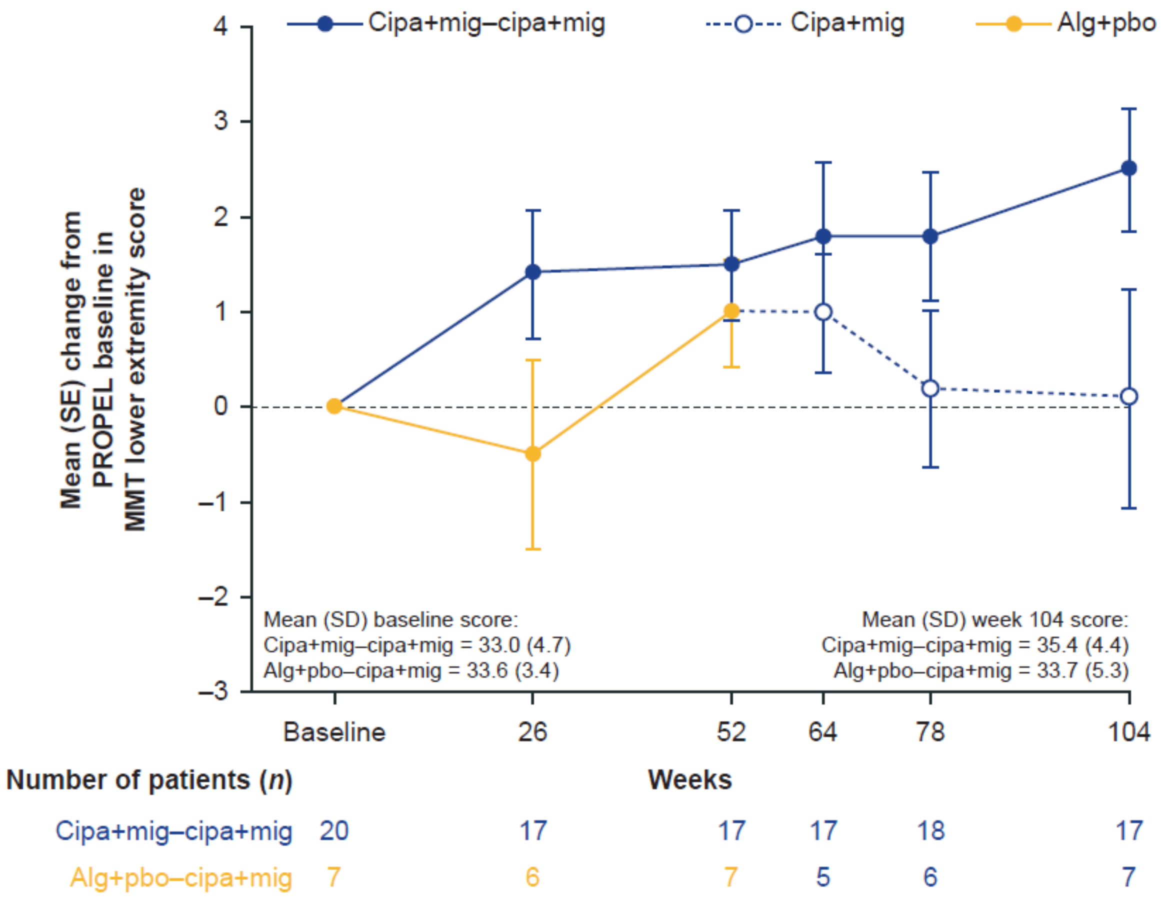

Supplementary Fig. S5 Change from baseline in MMT lower extremity score for ERT-naïve patients (OLE-ES population excluding outlier). *Alg+pbo* alglucosidase alfa+placebo; *cipa+mig* cipaglucosidase alfa+miglustat; *ERT* enzyme replacement therapy; *MMT* manual muscle testing; *OLE-ES* open-label extension enrolled subjects; *SD* standard deviation; *SE* standard error

a

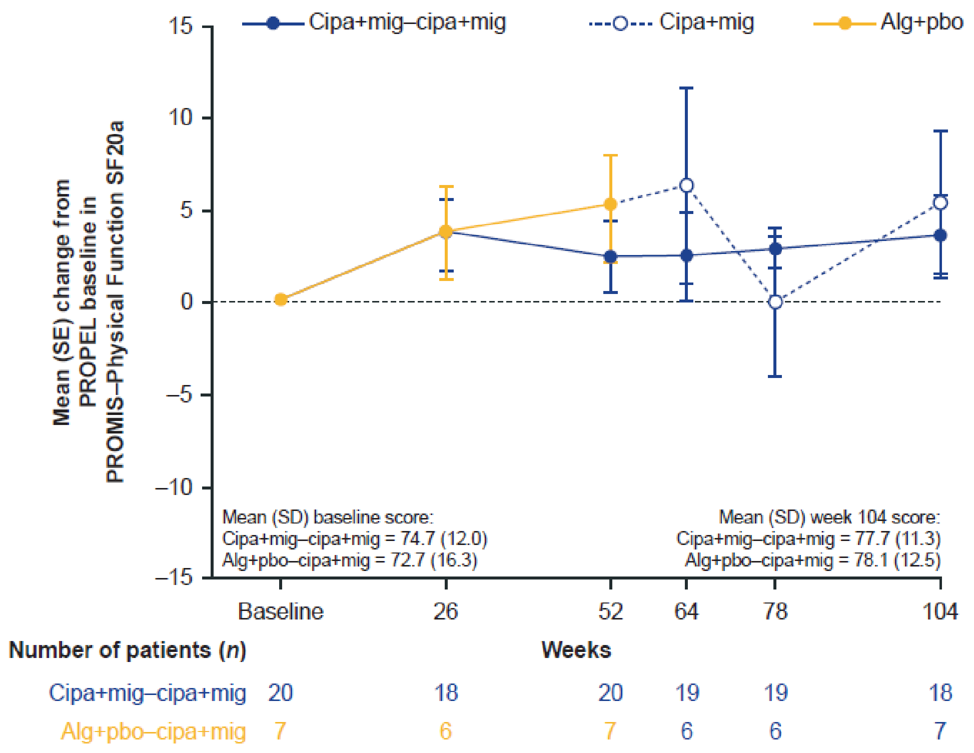

Cipa+mig–cipa+mig
Cipa+mig
Alg+pbo
Mean (SE) change from PROPEL baseline in PROMIS–Physical Function SF20a
15
10
5
0
–5
–10
–15
Mean (SD) baseline score:
Cipa+mig–cipa+mig = 74.7 (12.0)
Alg+pbo–cipa+mig = 72.7 (16.3)
Mean (SD) week 104 score:
Cipa+mig–cipa+mig = 77.7 (11.3)
Alg+pbo–cipa+mig = 78.1 (12.5)
Baseline
26
52
64
78
104
Weeks
Number of patients (n)
Cipa+mig–cipa+mig 20 18 20 19 19 18
Alg+pbo–cipa+mig 7 6 7 6 6 7

b

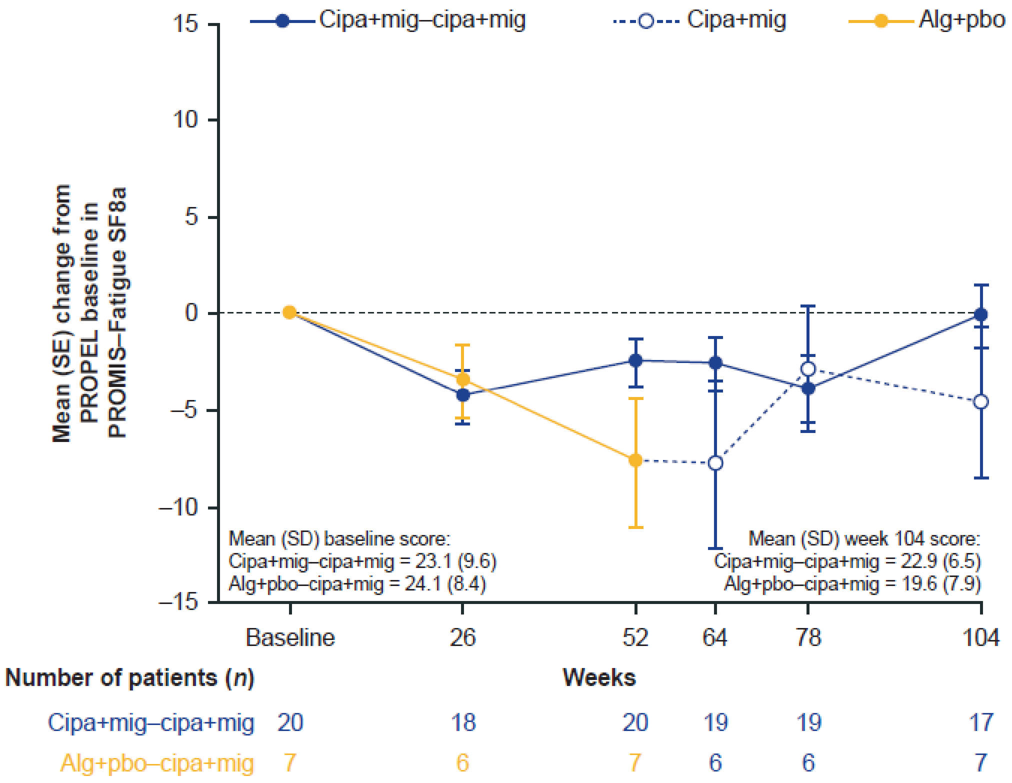

Cipa+mig–cipa+mig
Cipa+mig
Alg+pbo
Mean (SE) change from PROPEL baseline in PROMIS–Fatigue SF8a
15
10
5
0
–5
–10
–15
Mean (SD) baseline score:
Cipa+mig–cipa+mig = 23.1 (9.6)
Alg+pbo–cipa+mig = 24.1 (8.4)
Mean (SD) week 104 score:
Cipa+mig–cipa+mig = 22.9 (6.5)
Alg+pbo–cipa+mig = 19.6 (7.9)
Baseline
26
52
64
78
104
Weeks
Number of patients (n)
Cipa+mig–cipa+mig 20 18 20 19 19 17
Alg+pbo–cipa+mig 7 6 7 6 6 7

Supplementary Fig. S6 Change from baseline in PROMIS–Physical Function SF20a (a) and PROMIS–Fatigue score SF8a (b) for ERT-naïve patients (OLE-ES population excluding outlier). *Alg+pbo* alglucosidase alfa+placebo; *cipa+mig* cipaglucosidase alfa+miglustat; *ERT* enzyme replacement therapy; *OLE-ES* open-label extension enrolled subjects; *PROMIS* Patient-Reported Outcomes Measurement Information System; *SD* standard deviation; *SE* standard error
