# Supplementary material for: 104-week efficacy and safety of cipaglucosidase alfa plus miglustat in adults with late-onset Pompe disease: a phase III open-label extension study (ATB200-07)
Source: J Neurol. 2024 Feb 28;271(5):2810–23. doi: 10.1007/s00415-024-12236-0 (PMC11055775; doi:10.1007/s00415-024-12236-0)
Supplement: Supplementary file 2 — Supplementary file2 (PDF 1757 KB) [file 415_2024_12236_MOESM2_ESM.pdf]

104-week efficacy and safety of cipaglucosidase alfa plus miglustat in adults with late-onset Pompe disease: a phase III open-label extension study (ATB200-07)

Benedikt Schoser et al

What is Pompe disease?

Pompe disease is a rare, inherited disorder caused by **the lack of an enzyme called acid α-glucosidase (GAA)**, which is typically found inside muscle cells.

In healthy muscle cells, the **GAA enzyme breaks down the sugar glycogen into glucose.**

In **Pompe disease**, the lack of GAA enzyme activity means that **glycogen cannot be broken down** and **builds up inside muscle cells.** The damage this causes leads to **muscle weakness** and **breathing difficulties** over time.

Why did we do this study?

Treatment considerations

Currently, Pompe disease is treated with enzyme replacement therapies (ERTs). These therapies aim to replace the missing GAA enzyme in the muscle cells. However, there are several key challenges to consider when using ERTs in Pompe disease:

ERTs are unstable in the bloodstream

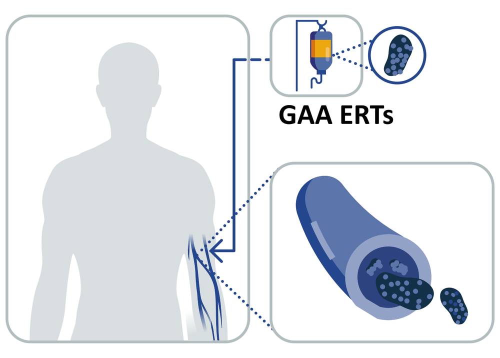

Efficient uptake into muscle and conversion into the most active form of GAA is required

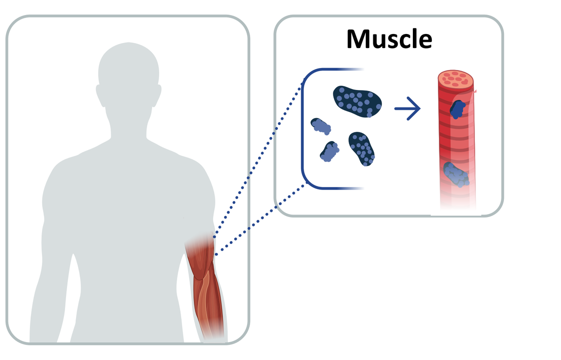

New investigational therapy: cipaglucosidase alfa + miglustat

Cipaglucosidase alfa + miglustat is a new two-component therapy that aims to:

Minimize breakdown of the ERT in the bloodstream before it reaches the target muscle cells

Improve uptake into muscle cells where cipaglucosidase alfa can work like the missing GAA

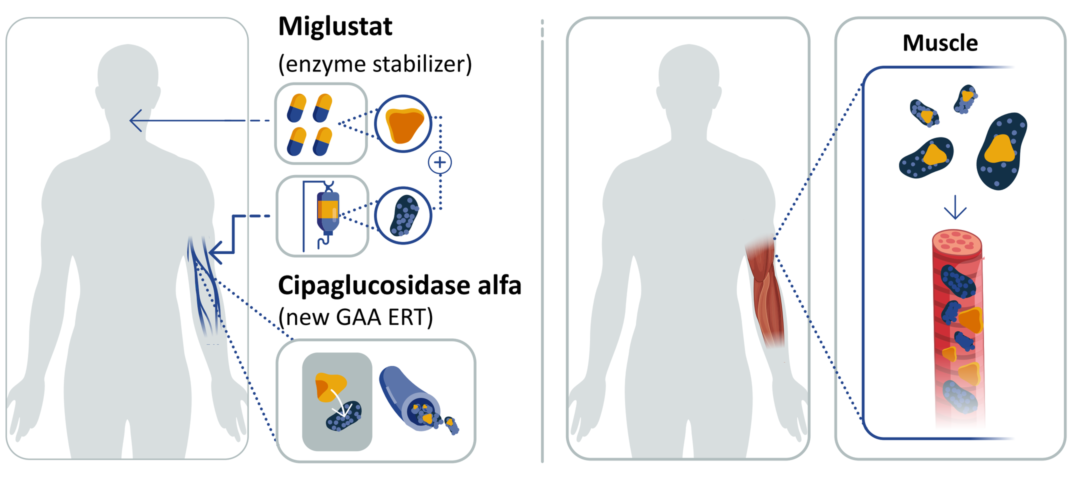

An ongoing extension study is investigating whether long-term treatment with cipaglucosidase alfa + miglustat improves the measurements of disease progression in people living with late-onset Pompe disease

How did we do this study?

In this extension study, all participants received cipaglucosidase alfa + miglustat every 2 weeks

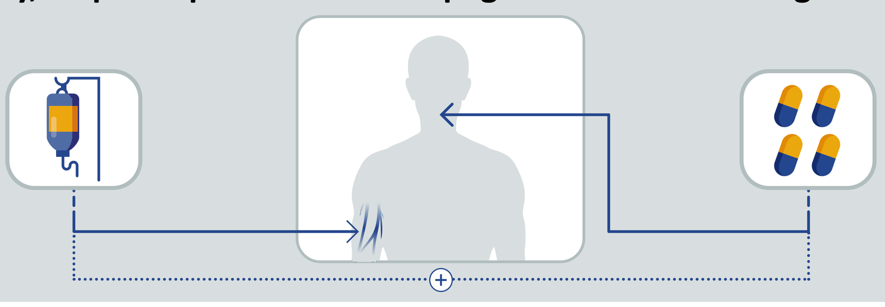

In a **previous trial, PROPEL,[1]** participants were treated for 52 weeks with cipaglucosidase alfa + miglustat or the current standard ERT, alglucosidase alfa + placebo

Group 1
Cipaglucosidase alfa + miglustat
n = 85

Group 2
Alglucosidase alfa + placebo
n = 38

In our **ongoing extension study,** we invited people who completed PROPEL to receive further treatment with, or switch to, cipaglucosidase alfa + miglustat

82 participants continued cipaglucosidase alfa + miglustat

37 participants switched treatment

All participants
Cipaglucosidase alfa + miglustat
n = 119

After 52 weeks in the extension study, participants were evaluated based on which treatment they received in PROPEL, and whether they had been treated with standard ERT before they started PROPEL

- **Group 1:** ERT experienced (*n* = 62)
- **Group 2:** ERT experienced (*n* = 29)
- **Group 1:** ERT naïve (*n* = 20)
- **Group 2:** ERT naïve (*n* = 8)

We checked standard Pompe disease assessments in participants at regular time intervals

% predicted[a] 6-minute walking distance (6MWD)
To assess **motor function**, we measured how far participants could walk in 6 minutes

% predicted[a] forced vital capacity (FVC)
To assess **lung function**, we used a spirometer to measure participants' FVC, a measure of how much air can be expelled from the lungs after taking a deep breath

Markers of muscle damage (CK)[2] and glycogen levels (Hex4)[3]
We measured levels of CK in blood and Hex4 in urine. Higher levels of CK and Hex4 typically indicate more muscle damage and higher glycogen build-up[2,3]

Safety profile
The **safety** of the study treatment was evaluated on an ongoing basis by closely monitoring participants for any medical issues

[a]6MWD and FVC are calculated as percent (%) predicted, which standardizes the results based on gender, age, height and weight for 6MWD, and gender, age, height and race for FVC

CK, creatine kinase; Hex4, hexose tetrasaccharide

- We also looked at other measurements of motor function and muscle strength, and scores given by the participants that assess their own quality of life (patient-reported outcomes)
- The results include measurements taken before treatment began in PROPEL (baseline), at the end of PROPEL (52 weeks) and at the 52-week mark of the extension study (104 weeks)
- This allowed us to investigate how treatment with cipaglucosidase alfa + miglustat impacts outcomes over an extended period of time

What have we found so far?

Group 1: participants who received cipaglucosidase alfa + miglustat for 104 weeks (in PROPEL and the extension study)

6MWD: a measure of motor function

ERT-experienced participants improved in 6MWD after 52 weeks of treatment with cipaglucosidase alfa + miglustat, and were then stable up to week 104

ERT-naïve participants also improved in 6MWD after 52 weeks of treatment with cipaglucosidase alfa + miglustat, and then improved further up to week 104

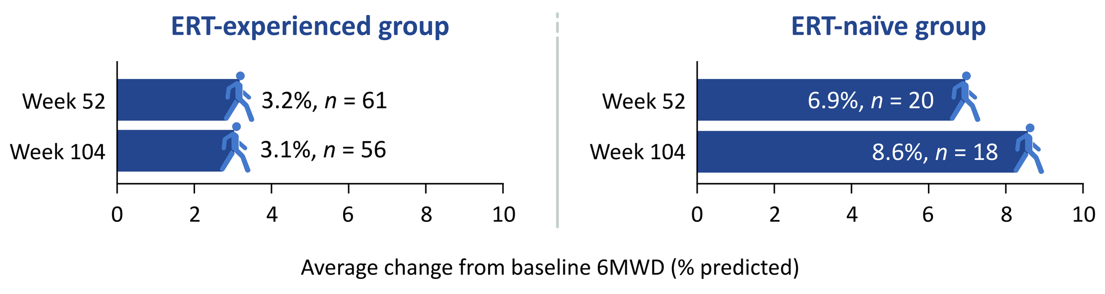

FVC: a measure of lung function

ERT-experienced participants' lung function over 104 weeks, throughout PROPEL and the extension study

ERT-naïve participants showed some decline in lung function worsened over the first 52 weeks of treatment in PROPEL but did not get worse during the extension study

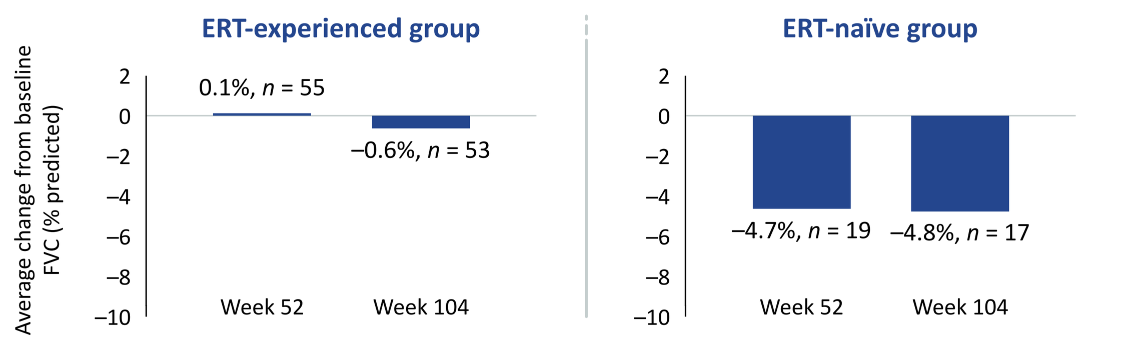

Group 2: participants who received alglucosidase alfa + placebo for 52 weeks in PROPEL and then switched to cipaglucosidase alfa + miglustat in the extension study

6MWD: a measure of motor function

ERT-experienced participants were stable in 6MWD after 52 weeks of treatment with alglucosidase alfa + placebo in PROPEL. Stability was maintained after switching treatment to cipaglucosidase alfa + miglustat

ERT-naïve participants showed improvements in 6MWD after 52 weeks of treatment with alglucosidase alfa + placebo. After switching to cipaglucosidase alfa + miglustat, 6MWD improved further to week 104

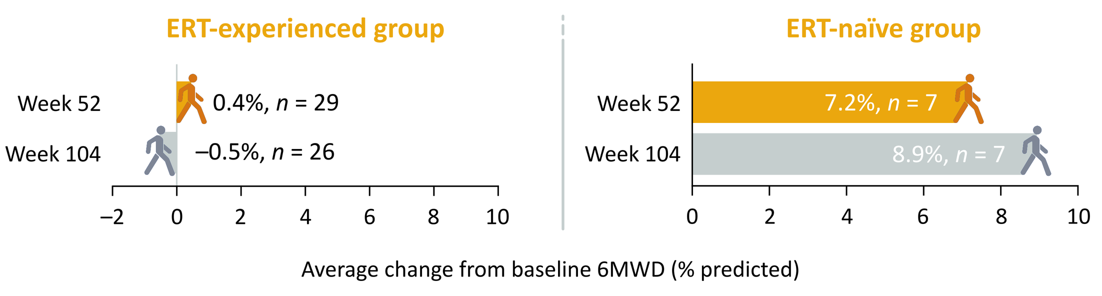

FVC: a measure of lung function

ERT-experienced and ERT-naïve participants had some decline in lung function during the 52 weeks of treatment with alglucosidase alfa + placebo in PROPEL. They then stabilized after switching to cipaglucosidase alfa + miglustat in the extension study up to week 104

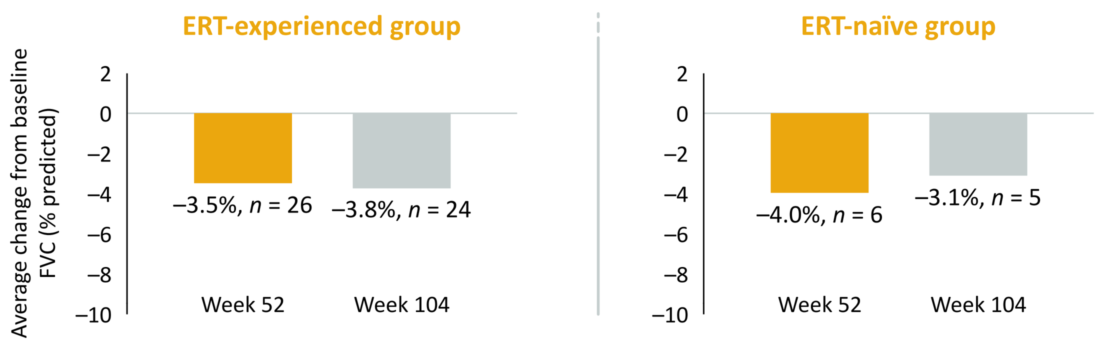

Blood levels of CK: a marker for muscle damage

CK levels improved from baseline in ERT-experienced and ERT-naïve participants treated with cipaglucosidase alfa + miglustat. By week 104, participants who switched treatment from alglucosidase alfa + placebo had similar CK levels to those treated with cipaglucosidase alfa + miglustat from baseline

Lower CK levels may indicate less damage to muscles

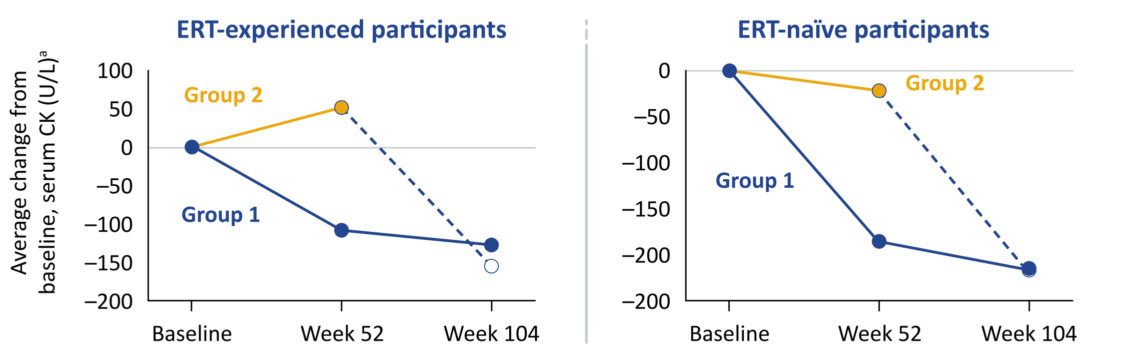

Group 1: cipaglucosidase alfa + miglustat in PROPEL and the extension study
Group 2: alglucosidase alfa + placebo in PROPEL; cipaglucosidase alfa + miglustat in the extension study

[a]Measured in units per liter of blood serum (U/L)

Urine levels of Hex4: a marker for glycogen build-up

Hex4 levels in both ERT-experienced and ERT-naïve participants treated with cipaglucosidase alfa + miglustat improved from baseline. By week 104, participants who switched treatment from alglucosidase alfa + placebo had similar Hex4 levels to those treated with cipaglucosidase alfa + miglustat from baseline

Lower Hex4 levels indicate that the treatment may be breaking down glycogen in muscle cells

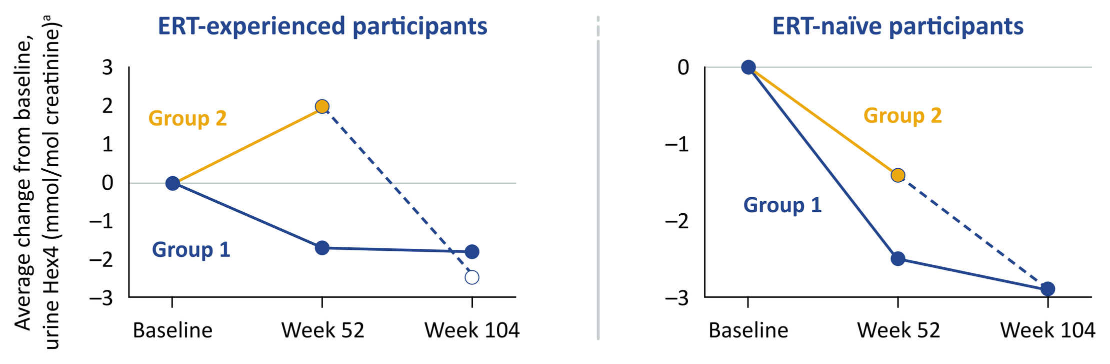

Group 1: cipaglucosidase alfa + miglustat in PROPEL and the extension study
Group 2: alglucosidase alfa + placebo in PROPEL; cipaglucosidase alfa + miglustat in the extension study

[a]Measured in comparison to the amount of a key protein in urine (mmol/mol creatinine)

Safety profile

Most medical issues experienced by participants were **mild or moderate** in severity and three led to withdrawal of the participants from the study

Medical issues in all participants receiving cipaglucosidase alfa + miglustat in PROPEL and the extension study (*n* = 122)

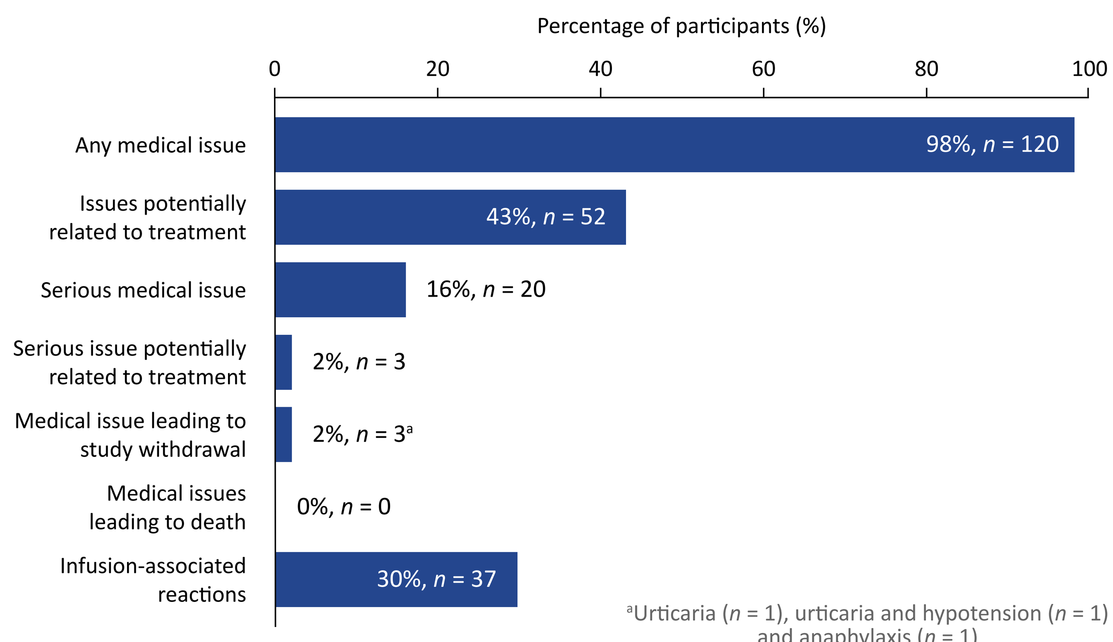

[a]Urticaria (*n* = 1), urticaria and hypotension (*n* = 1) and anaphylaxis (*n* = 1)

The most common **medical issues** that occurred after participants started the study treatment were:[b]

- **Fall** 39%, *n* = 48
- **Headache** 34%, *n* = 41
- **Joint pain** 30%, *n* = 37
- **Muscle pain** 25%, *n* = 30
- **Common cold** 20%, *n* = 25

[b]% calculated from all participants in PROPEL and the extension study (*n* = 122)

What do our results mean for people with late-onset Pompe disease?

Participants treated with cipaglucosidase alfa + miglustat had overall improvement or stability in % predicted 6MWD, % predicted FVC and biomarker levels over the course of the extension study

Participants **could walk further or a similar distance in 6 minutes** over the course of the extension study. This indicates that the **motor function** of participants **improved or stabilized**

The **lung function** of participants **stabilized** during the extension study. This indicates that, on average, participants' **breathing did not get worse**

A blood **marker for muscle damage (CK)** and a urine **marker for glycogen build-up (Hex4)** both decreased, which could indicate **less muscle damage,** potentially because of **a reduction in disease progression**

Most **medical issues** were **mild or moderate in severity**. Few participants stopped treatment because of medical issues. The overall safety profile of cipaglucosidase alfa + miglustat was consistent with currently available ERTs

This summary accompanies the manuscript titled '104-week efficacy and safety of cipaglucosidase alfa plus miglustat in adults with late-onset Pompe disease: a phase III open-label extension study (ATB200-07)'.

[1]Schoser B et al (2021) Lancet Neurol 20:1027–1037; [2]Brancaccio P et al (2010) Clin Chem Lab Med 48:757–767; [3]An Y et al (2005) Mol Genet Metab 85:247–254.

Clinical trial identifier: NCT04138277.
This study was supported by Amicus Therapeutics, Inc., Princeton, NJ, USA.
Medical writing assistance was provided by Kara Filbey, PhD, at Cence (an AMICULUM® agency), funded by Amicus Therapeutics, Inc.
